# Microfluidic Organoid Cultures Derived from Pancreatic Cancer Biopsies for Personalized Testing of Chemotherapy and Immunotherapy

**DOI:** 10.1002/advs.202303088

**Published:** 2023-11-29

**Authors:** Daheui Choi, Alan M. Gonzalez‐Suarez, Mihai G. Dumbrava, Michael Medlyn, Jose M. de Hoyos‐Vega, Frank Cichocki, Jeffrey S. Miller, Li Ding, Mojun Zhu, Gulnaz Stybayeva, Alexandre Gaspar‐Maia, Daniel D. Billadeau, Wen Wee Ma, Alexander Revzin

**Affiliations:** ^1^ Department of Physiology and Biomedical Engineering Mayo Clinic Rochester MN 55905 USA; ^2^ Division of Experimental Pathology Mayo Clinic Rochester MN 55905 USA; ^3^ Center for Individualized Medicine Epigenomics program Mayo Clinic Rochester MN 55905 USA; ^4^ Division of Oncology Research College of Medicine Mayo Clinic Rochester MN 55905 USA; ^5^ Department of Medicine University of Minnesota Minneapolis MN 55455 USA; ^6^ Division of Medical Oncology Mayo Clinic Rochester MN 55905 USA

**Keywords:** chemotherapy, immunotherapy, microfluidic device, pancreatic cancer, patient‐derived organoid

## Abstract

Patient‐derived cancer organoids (PDOs) hold considerable promise for personalizing therapy selection and improving patient outcomes. However, it is challenging to generate PDOs in sufficient numbers to test therapies in standard culture platforms. This challenge is particularly acute for pancreatic ductal adenocarcinoma (PDAC) where most patients are diagnosed at an advanced stage with non‐resectable tumors and where patient tissue is in the form of needle biopsies. Here the development and characterization of microfluidic devices for testing therapies using a limited amount of tissue or PDOs available from PDAC biopsies is described. It is demonstrated that microfluidic PDOs are phenotypically and genotypically similar to the gold‐standard Matrigel organoids with the advantages of 1) spheroid uniformity, 2) minimal cell number requirement, and 3) not relying on Matrigel. The utility of microfluidic PDOs is proven by testing PDO responses to several chemotherapies, including an inhibitor of glycogen synthase kinase (GSKI). In addition, microfluidic organoid cultures are used to test effectiveness of immunotherapy comprised of NK cells in combination with a novel biologic. In summary, our microfluidic device offers considerable benefits for personalizing oncology based on cancer biopsies and may, in the future, be developed into a companion diagnostic for chemotherapy or immunotherapy treatments.

## Graphical abstract

Treatment of pancreatic cancer may be made more effective by selecting therapies for an individual patient. The authors developed and characterized microfluidic devices that may be used to test chemotherapies and immunotherapies using needle core biopsies from patients with pancreatic cancer.

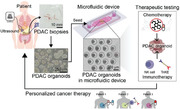

## 1 Introduction

There is an increasing realization that one‐size‐fits‐most approaches to treating cancer patients are ineffective and that treatment regimens need to be personalized to an individual patient. This necessitates the development of patient‐specific cancer models for therapy testing.^[^ ^1^ ^]^ Patient‐derived cell lines and patient‐derived xenograft (PDX) models represent some of the established approaches for individualizing cancer therapy selection.^[^ ^2^ ^]^ While incredibly useful, these strategies have disadvantages. Cell lines undergo genetic and morphological changes over time in culture, resulting in inconsistent and inaccurate outcomes.^[^ ^3^ ^]^ PDX models take several months to establish with success rates for tumor engraftment varying from 20 to 60% depending on the tumor type.^[^ ^4^ ^]^ PDX models of pancreatic ductal adenocarcinoma (PDAC) are challenging to establish given the high degree of fibrosis and low cellularity of PDAC tissue.^[^ ^5^ ^]^ Furthermore, PDAC is an aggressive cancer with 65% of diagnoses occurring at stage III or higher with median survival time of 8–10 months.^[^ ^6^ ^]^ The time required to establish a PDX model often approaches the survival time which means that a given model may not be used for personalized therapy.

Patient‐derived cancer organoids (PDOs) have the potential of addressing limitations of other patient‐specific cancer models.^[^ ^2a^ ^]^ PDOs are 3D self‐organizing structures that are derived from primary tumor cells^[^ ^7^ ^]^ and provide dimensionality and topology similar to that of the native tumor.^[^ ^2a^ ^]^ Compared to other cancer models, PDOs are less time‐intensive and costly to establish and may therefore be used to rapidly select suitable drugs for aggressive cancers. PDAC organoid cultures were first described by Muthuswamy and Tuveson labs in 2015.^[^ ^8^ ^]^ Since then, PDOs derived from PDAC and other tumor types have been shown to recapitulate patient‐specific abnormalities and to be predictive of patient responses to therapy.^[^ ^9^ ^]^

Biopsies are routinely collected during diagnosis of PDAC and represent starting material for PDO generation.^[^ ^10^ ^]^ However, needle core biopsies typically have a low number of cells (≈100000–500000 cells depending on the cancer type and patient),^[^ ^11^ ^]^ which makes it challenging and time‐consuming to generate organoids in sufficient numbers to test therapies using standard culture systems.^[^ ^12^ ^]^ The time window between diagnosis of PDAC and chemotherapy administration is ≈4 weeks which means that organoid formation, expansion, and therapy testing needs to fit within that time frame.

Microfluidic cell cultures are particularly well‐suited for scenarios where testing of multiple drugs needs to be carried out with a limited number of cells for cultivating cancer cells and testing responses to therapy.^[^ ^13^ ^]^ For example, Prince et al. developed a microfluidic platform integrating gradient generators and hydrodynamic traps and used this device to culture clusters of cancer cells and test their response to different concentrations of drugs.^[^ ^13c^ ^]^ The Takayama lab has developed a microfluidic hanging drop system for culturing cancer spheroids and testing chemotherapies,^[^ ^13d^ ^]^ while the Kamm lab has pioneered microfluidic cultures integrating cancer cells with vasculature to study extravasation.^[^ ^14^ ^]^ There have also been reports of microfluidic cancer cultures for testing cancer immunotherapy.^[^ ^15^ ^]^ For example, Ayuso et al. employed a tumor‐on‐chip system to explore anti‐cancer activity of NK cells,^[^ ^15a^ ^]^ while Ronteix et al. used a droplet microfluidic device to co‐entrap cancer and immune cells for testing immunotherapies.^[^ ^15b^ ^]^ Additional approaches employed microcapsules comprised of extracellular matrix (ECM) and carrying cancer spheroids for therapy testing. For example, Ding et al. demonstrated that colon cancer spheroids cultured in Matrigel microparticles retained cellular heterogeneity of native tumor tissue, could be used to test immunotherapies, and were predictive of patient responses to chemotherapy.^[^ ^3^ ^]^ With a few recent exceptions,^[^ ^3^, ^16^ ^]^ microfluidic cultures reported to date relied on cancer cell lines or PDX models. To the best of our knowledge, there has not been a report of microfluidic organoid cultures derived from patient biopsies in general and from PDAC biopsies specifically.

We have previously demonstrated that microfluidic devices are well‐suited for rapidly testing therapies based on an input of small number of cells.^[^ ^17^ ^]^ In this prior study, ovarian cancer organoids were derived from PDX tissue and shown to better proliferate and maintain epithelial cancer phenotype in microfluidic cultures compared to traditional culture approaches (standard 3D plate and Matrigel cultures).

**Figure 1:**
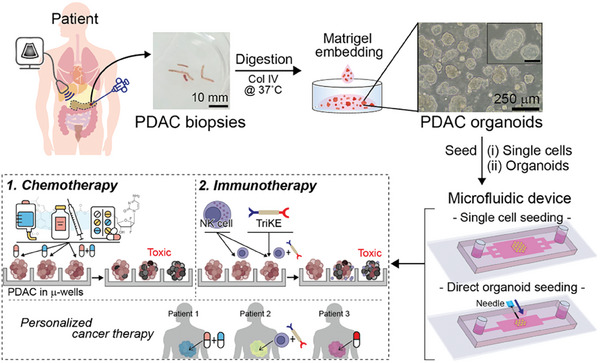
Schematic describing the concept of this study: Needle core biopsies were collected from patients, digested, cultured as organoids, and then placed into microfluidic devices. Two types of microfluidic devices were used in this study: 1) device type 1 for seeding single cells and 2) device type 2 for seeding intact organoids or organoid fragments. Microfluidic organoid cultures were used to test several chemotherapy drugs as well as a novel immunotherapy comprised of NK cells and immunomodulator molecule TriKE.

In the present study, we take the concept of microfluidic cultures farther and describe microfluidic cultivation of PDAC organoids derived from patient needle biopsies. As shown in **Figure** **1**, our devices contained arrays of microwells (250 µm diameter) that were made low‐binding to promote aggregation of cells into spheroids. To address the challenge of minimal number of cells available from biopsies, our novel microfluidic device incorporated a port for direct injection of organoids or organoid fragments. This design feature enhanced efficiency of organoid/tissue utilization. Our study demonstrates that while microfluidic organoids were comparable to the gold‐standard Matrigel‐based organoids in terms of gene and phenotype expression, microfluidic cultures offered multiple advantages including 1) uniform size and ease of assessing effects of chemotherapy, 2) the ability to add NK cells to create immune cell‐cancer organoid co‐cultures and to study cancer‐immune cell interactions, and 3) minimal reliance on Matrigel. In addition, to demonstrating cultivation of three different PDO lines in the novel device we also assessed novel therapies. These included GSK inhibitor therapy which was shown to sensitize PDO responses to front‐line chemotherapy (gemcitabine) and also cancer immunotherapy consisting of NK cells and a novel immunomodulatory biotherapeutic. Overall, our study highlights utility of microfluidic organoid cultures for assessing cancer treatment using scant tissue available from pancreatic cancer biopsies and represents a step toward establishing these cultures as a companion diagnostic in the future.

## 2 Results and Discussions

### 2.1 Comparing Microfluidic and Standard Organoid Cultures

**Figure 2:**
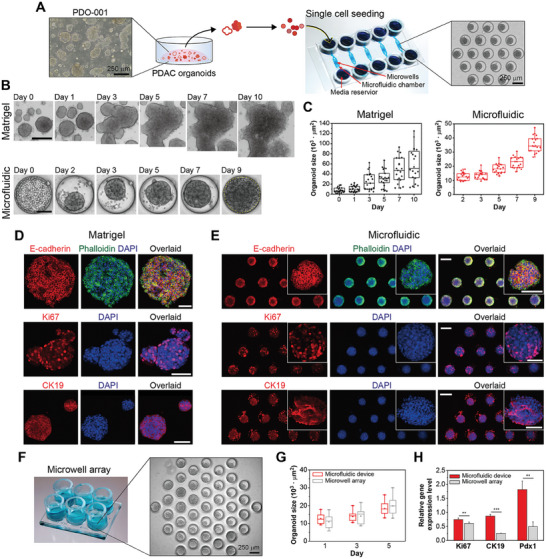
Formation of PDAC organoids in microfluidic devices using single cell digest as starting material. A) Illustration of single cell seeding in microwells from PDOs. Microfluidic device type 1 used to form organoids from single‐cell suspension. Inset image shows organoids (PDO‐001) in microfluidic device after 5 days of seeding. B) Images of organoids from PDO‐001 in Matrigel (upper panel) and microfluidic device (lower panel) at different time points during culture (Scale bars = 100 µm). C) Quantification of organoid size (area) change in Matrigel and microfluidic devices. D and E) Immunofluorescence staining for epithelial (E‐cadherin), PDAC (CK19), and proliferation (Ki67) markers in organoids cultured in Matrigel D) and microfluidic devices E). The scale bars are 100 µm for Matrigel and magnified inset images in microwell, and 200 µm for microarray. F) Microwell array culture format—cloning cylinders were mounted onto a PDMS based with arrays of microwells. Inset image showing organoids (PDO‐001) at 5 days of culture. G) Comparing organoid growth in microwell array and microfluidic cultures. Merging of organoids made it challenging to quantify growth rates in Matrigel cultures. H) Comparing gene expression of proliferation marker (Ki67) and cancer markers (CK19 and Pdx1) for culture formats. Statistical significance of each group indicates ** for *p* < 0.01 and *** *p* < 0.001, respectively.

One key objective for this study was to evaluate novel microfluidic organoid cultures in comparison to more standard culture formats—Matrigel and microwell arrays. Throughout this study we used two variants of microfluidic devices—device 1 for seeding single cells in scenarios with relative abundance of cells and device 2 for seeding intact organoids or organoid fragments when cells were scant. Comparison of different culture formats reported below was carried out with PDO‐001—a poorly differentiated cancer that proliferated in vitro producing cells in numbers sufficient for in‐depth analysis. **Figure** **2**A and S1 (Supporting Information) shows a workflow for the study—PDAC biopsies were dissociated, expanded as organoids on Matrigel, and then collected and seeded into microfluidic devices. Figure S2 (Supporting Information) shows images of patient biopsies used in this study. The extent of expansion on Matrigel was variable. Ten to twenty passages were possible for PDO‐001 ensuring supply of these organoids while only five to seven passages were possible for PDO‐002 and ‐003. Thus, the latter two tissues were of limited supply and were cultured in device 2.

Figure 2A shows device 1 that consisted of five independent microfluidic compartments, each with media reservoirs and transport channels passively delivering nutrients to the cell culture chamber. The latter contained an array of 19 wells, each of 250 µm in diameter. The devices were composed of gas‐permeable silicone rubber (PDMS) allowing diffusion of atmospheric oxygen and were functionalized with nonionic surfactant Pluronic to make microwells low‐binding and to promote spheroid formation.^[^ ^18^ ^]^ As may be appreciated from the images in Figure S3 (Supporting Information) and Figure 2B, spheroid formation occurred 2 days after seeding, with spheroids increasing in diameter during subsequent 7 days of culture. Compared to Matrigel cultures, spheroids in microfluidic devices were more uniformly distributed (Figure 2C and Figure S3, Supporting Information). This is a key advantage of our culture format, making it possible to observe changes in spheroid geometry over time in the presence or absence of therapy. Comparable tracking is challenging with Matrigel cultures because spheroids are not uniform and have a tendency to merge (Figure 2B; Matrigel).

In an effort to benchmark microfluidic organoids against the gold‐standard Matrigel cultures, we carried out immunofluorescence staining of both culture formats. These results, shown in Figure 2D,E, demonstrate similarities between the two cultures. Both stained strongly for E‐cadherin – a cell adhesion marker indicative of epithelial cells comprising pancreatic ducts.^[^ ^19^ ^]^ Microfluidic (inset) and Matrigel organoid cultures also had comparable expression levels of a PDAC‐specific marker CK19,^[^ ^20^ ^]^ and a proliferation marker Ki67.

There have been several reports of microwell array systems used for creating uniformly sized cancer organoids and testing therapies.^[^ ^21^ ^]^ Therefore, we wanted to set up a microwell array as another comparator culture format and fabricated PDMS insert with an array of microwells of the same diameter and pitch as the microwells of the microfluidic device. However, unlike the microfluidic device where spheroids were confined to a local volume of 3 μl, the microwells in the insert were placed into 150 µL – a volumetric equivalent of a well from a 96‐well plate. This experiment was designed to address a question of whether small local volume of the microfluidic device was beneficial for proliferation and phenotype maintenance of pancreatic cancer organoids. Previously, we demonstrated this to be the case for ovarian cancer organoids that proliferated and expressed epithelial cancer phenotype better in microfluidic devices compared to microwell arrays.^[^ ^17^ ^]^ The custom microwell arrays used for comparison with microfluidic cultures are shown in Figure 2F. Comparison of changes in organoid size over time revealed similar proliferation dynamics for microfluidic device and microwell array (see Figure 2G); however, PCR analysis highlighted better gene expression in the microfluidic device. As seen from Figure 2H, PDAC organoids in the microwell array cultures were significantly less proliferative (lower Ki67) and expressed lower levels of PDAC‐specific markers, CK19 and Pdx1 compared to microfluidic organoid cultures. Given better expression of proliferation and phenotypic markers, we relied exclusively on the microfluidic organoid cultures for testing chemotherapies and immunotherapy going forward.

**Figure 3:**
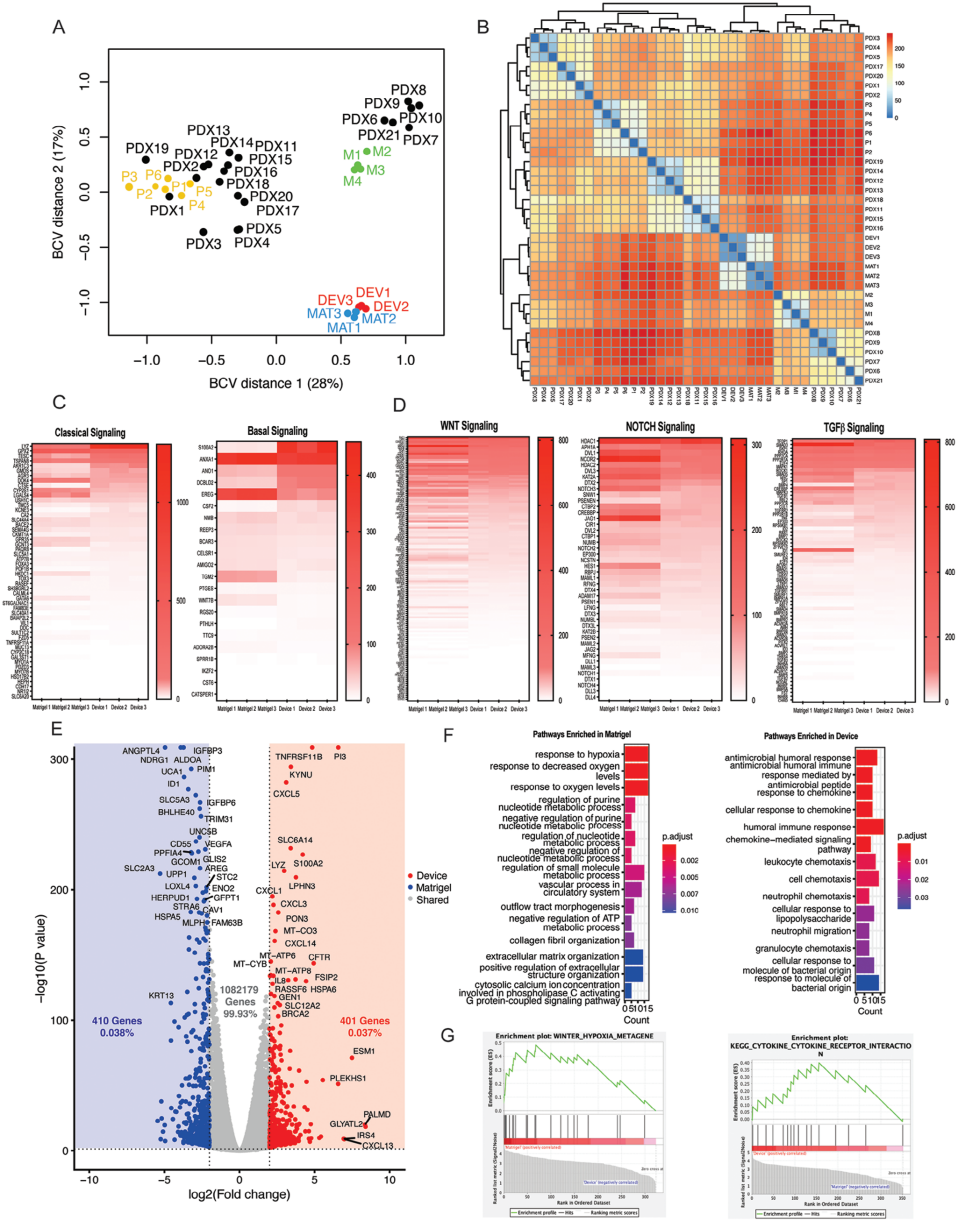
Transcriptomic comparison of Matrigel‐based and microfluidic organoid cultures. A) Multidimensional scaling (MDS) plot comparing PDOs in microfluidic devices and Matrigel cultures, and with publicly available organoid sequencing data from primary PDAC tumors or P (*n* = 6), PDXs (*n* = 21), and metastases or M (*n* = 4). Five hundred top expressed genes from each sample were used to calculate pairwise distances between samples. B) Heatmap of sample‐to‐sample distances via Pearson correlation between microfluidic culture compared to Matrigel culture and to PDOs. C) Heatmaps of normalized gene expression counts (reads per kilobase per million mapped reads or rpkm) for Basal and classical PDAC subtype markers in PDOs cultured in devices versus Matrigel. D) Heatmaps of normalized gene expression counts (rpkm) for WNT, NOTCH, and TGF Beta signaling pathways. E) Volcano plot with genes significantly upregulated and downregulated between PDOs in devices compared to Matrigel culture highlighted. The horizontal line corresponds to ‐log_10_(*p*‐value) with *p*‐value = 0.05 and the two vertical lines represent log_2_(fold change) of 2 and −2. Hits were selected to have an ‐log_10_(0.05) and effect size > 2 or < −2. F) Gene Ontology of biological processes enriched in the Matrigel culture and Microfluidic culture differentially expressed genes. G) Selected GSEA enrichment plots highlighting key pathways enriched in Matrigel and microfluidic culture platforms. Top 20 GSEA enrichment plots for Matrigel and microfluidic culture format may be found in Figures S5,S6 (Supporting Information) respectively.

Having demonstrated benefit of maintaining organoids in microfluidic devices over microwell arrays, we proceeded with a more in‐depth comparison of Matrigel and microfluidic organoid cultures. Culturing PDAC organoids in Matrigel in WRN‐containing media was first described by Boj et al. in 2015 remains the gold standard today.^[^ ^22^ ^]^ We carried out RNA sequencing of organoids derived from the same patient biopsy (PDO‐001) after cultivation in either Matrigel or microfluidic devices. The study was limited to one patient because of the scarcity of organoids available from other patients (PDO‐002 and −003). First, samples were compared to each other and to an existing dataset of publicly available samples from Hogenson et al.^[^ ^9c^ ^]^ As shown in **Figure** **3**A and Figure S4 (Supporting Information), the microfluidic device (Device) and Matrigel samples clustered closer to each other and were separated by one principal component accounting for 74% of the distance between the samples (Figures 3A and Figure S4, Supporting Information). The device and the Matrigel organoids were more similar to each other than to other PDAC PDO samples generated from primary tumors, PDX and metastases on principal component analysis and when comparing sample to sample distances (Figure 3A,B). This was reflected by similar gene expression of classical and basal pancreatic cancer signaling and developmental pathways (WNT, NOTCH and TGF‐β) known to be involved in organoid formation and maintenance (Figures 3C and 2D).^[^ ^23^ ^]^ Several genes associated with the Classical PDAC subtype (e.g., *LYZ* and *TESC)* and with the basal‐like subtype that is linked to worse outcomes (e.g., S100A2, ANO1, and DCBLD2) were upregulated in microfluidic devices. A volcano plot of the differentially expressed genes enriched in Matrigel and device organoid cultures is shown in Figure 3E and Figure S4C,D (Supporting Information). From this analysis, we determined that the majority of genes (99.93%) were similarly expressed between the device and Matrigel organoid cultures with only 0.038% genes enriched in Matrigel and 0.037% in the microfluidic device (Figure 3E). The full list of differentially expressed and shared genes between matrigel and microfluidic devices is in Table S1 (Supporting Information). Using gene ontology analysis for biological processes (Figure 3F) and gene set enrichment analysis against curated gene sets (Figure 3G), we determined that Matrigel organoid cultures were enriched for pathways related to hypoxia, which may point to insufficient oxygenation compared to microfluidic organoid cultures. Matrigel cultures were also enriched for changes in cell metabolism including the regulation of purine synthesis and ATP biosynthesis. These changes may suggest increased anaerobic respiration due to the hypoxic state of organoids in Matrigel and may negatively impact their long‐term viability, phenotype, and response to therapy. By contrast, microfluidic organoids appeared to be enriched in pathways related to improved communication with the microenvironment (Figure 3F,G). Of particular note was upregulated expression of cytokines known to promote chemotaxis of NK cells, including C─C motif chemokine ligand (CCL)‐5, CCL‐7, and C‐X‐C chemokine motif ligand (CXCL)‐14.^[^ ^24^ ^]^ Also enriched was the chemokine CXCL13 which has been shown to regulate lymphocyte infiltration within the tumor microenvironment.^[^ ^25^ ^]^ Our findings are significant for several reasons. First, microfluidic organoids resemble the gold‐standard Matrigel organoids in expression of genes associated with pancreatic development, organoid formation, and maintenance. This observation is significant because Matrigel is a key component of stem cell or cancer organoid culures^[^ ^26^ ^]^ and yet it was used very sparingly (1% in media for 2 days during spheroid formation) in the microfluidic cultures. Second, upregulated expression of genes associated with chemotaxis of leukocytes in general and NK cells specifically supports our hypothesis that small volumes of microfluidic devices amplify autocrine/paracrine signaling and points to the benefits of using microfluidic organoid cultures for testing cellular immunotherapy such as NK cell therapy described later. We remind a reader that practical benefits of microfluidic devices over Matrigel cultures extend beyond gene expression and include 1) uniform distribution of spheroid sizes, 2) ease of tracking individual organoids over time, and 3) the ability to create co‐cultures with immune cells.

### 2.2 Culturing Intact Organoids in a Microfluidic Device

**Figure 4:**
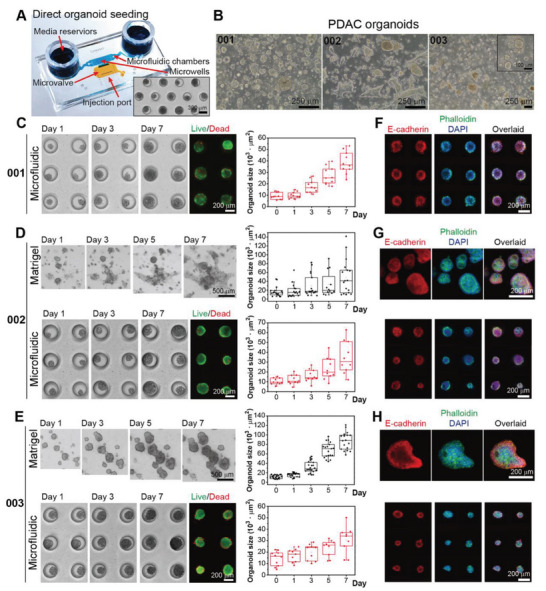
Microfluidic cultures of intact PDAC organoids A) A novel microfluidic device with an injection port used for direct placement of intact organoids into the culture chamber. Inset shows intact organoids (PDO‐001) after 5 days of culture. B) Representative images of organoids in Matrigel derived from three different patients. C–E) Time‐course images of organoids in Matrigel and microfluidic cultures. Live/Dead staining was carried out for microfluidic organoid cultures at day 7. F–H) Immunofluorescence staining for E‐cadherin (Red) for all three organoid lines in Matrigel and microfluidic devices. Green and blue staining is for actin (phalloidin) and nuclei (DAPI) respectively.

As noted earlier, PDAC biopsies often have low cellularity and present a distinct challenge for organoid formation and expansion. In the case of two patient biopsies, we found it is difficult to generate organoids in numbers sufficient for a microfluidic device type 1 described in Figure 2. Given significant losses of cellular material during organoid dissociation and filtration to produce single‐cell suspension, we wanted to modify the design of the microfluidic device to introduce intact organoids or organoid fragments (≈100‐ to 150‐µm diameter). This novel device (see **Figure** **4** **A**) had a side injection port protected by a normally closed valve. Negative pressure was applied to open the valve and a needle was introduced to transfer intact organoids directly into the culture chamber. We compared organoids from three different patients, denoted as PDO‐001, PDO‐002, and PDO‐003, in microfluidic devices and Matrigel cultures. Images of Matrigel cultures for all three PDOs are provided in Figure 4B. It may be appreciated that PDOs formed into uniform spheroids in the microfluidic device but were more randomly distributed in Matrigel cultures. In fact, no formation of distinct spheroids was observed for PDO‐002. Different patients were morphologically distinct. The organoids in PDO‐001 were compact, solid clusters with clear edges, whereas PDO‐003 had solid spherical structures with loose cells or debris accumulating around organoids over time. Cultures of PDO‐002 presented two distinct morphologies in Matrigel: spherical and elongated/stretched. Some of the organoids from PDO‐001 and ‐002 had luminal structures, while no such structures were observed for PDO‐003. Microfluidic organoid cultures exhibited features observed in Matrigel, with PDO‐001 and ‐002 exhibiting luminal/ductal structures (Figure 4C,D) and PDO‐003 forming compact spheroids (Figure 4E).

Next, we compared proliferation and phenotype expression for all three patient organoids in microfluidic and Matrigel cultures. As may be seen from Figure 4C to E, all three organoid types proliferated well in microfluidic devices albeit with different growth rates. These images also highlight challenges of characterizing organoids in Matrigel culture where individual clusters fused over time contributing to a wide distribution of organoid sizes. In contrast, organoids in microfluidic cultures had a much narrower size distribution. Growth rates and size distribution may be better appreciated in Figure 4C–E. The size changes over time for PDO‐001 cultured as intact spheroids in microfluidic devices were comparable to that of organoids in single‐cell devices (Figures 2C and 4C), demonstrating that these devices may be used interchangeably depending on cellularity and organoid expansion dynamics of individual tumor biopsies.

Importantly, microfluidic organoid cultures for all three patients stained strongly for epithelial marker E‐cadherin, PDAC marker CK19, and proliferation marker Ki67 (Figure 4F–H and Figures S7,S8, Supporting Information). Levels of expression for these markers were visually comparable to organoids in Matrigel. In summary, we demonstrated intact organoids derived from three different PDAC biopsies could be cultured in the novel microfluidic device with markers of proliferation and epithelial/cancer phenotype expression similar to the gold‐standard Matrigel organoid cultures.

### 2.3 Assessing Interactions of PDMS‐Based Microfluidic Devices with Chemotherapy Drugs

Our microfluidic devices are comprised of PDMS—material that has the potential to take up small hydrophobic molecules that are typically used as chemotherapy drugs.^[^ ^27^ ^]^ We wanted to assess the extent to which absorption of chemotherapy drugs occurred in our devices. To carry out this assessment, microfluidic devices were prepared in the way that was identical to cell culture experiments. The devices were functionalized with Pluronic (nonionic surfactant that imparts low‐binding property) and then incubated with serum‐containing media for 3 days. The latter step was designed to mimic 3‐day period allotted for organoid formation prior to drug testing. The devices did not contain cells to eliminate the possibility of drugs being taken up by cells and to focus squarely on PDMS uptake. Chemotherapy drugs (Encorafenib (En), Binimetinib (Bi), Gemcitabine (GEM), and GSKI) were prepared at concentrations ranging from 10 to 100 μM in media and were incubated in devices at 37 °C for 2 days. Identical protocol was followed for drug testing with PDOs. UV–Vis spectroscopy was used to construct absorbance versus concentration calibration curves for individual drugs (see Figure S9A, Supporting Information) and to determine drug concentration. As highlighted by the data in Figure S9B (Supporting Information), the uptake of chemotherapy drugs in PDMS devices was negligible. This observation may be attributed to a combination of factors including 1) hydrophilic functionalization of the surface with nonionic surfactant Pluronic and 2) potential deposition of serum proteins from the media. Modest increases in concentration for En and Bi are likely due to media evaporation during 2 days of incubation in microfluidic devices. Thus, our analysis shows that the chemotherapy drugs of interest to this study were not taken up by the PDMS‐based microfluidic devices and that drug concentrations experienced by PDOs in the devices were expected to be similar to the original concentrations.

### 2.4 Testing Molecular Therapies Targeting RAF and MEK Kinases Using Microfluidic Organoid Cultures

**Figure 5:**
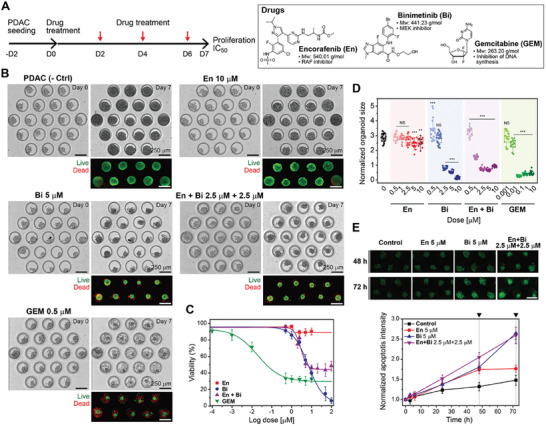
Chemotherapy targeting MEK and RAF signaling tested in microfluidic cultures. A) (Left) Experimental timeline for seeding cells and administering drugs. (Right) Chemical structures of drugs used in this experiment: En, Bi, and GEM. B) Representative images of microfluidic organoid cultures at day 0 and day 7 (end of treatment) for each group. The fluorescence images represent Live/Dead staining at day 7 (Green: Live, Red: Dead). C) Relative drug response graph for four drug treatment groups after 7 days of treatment (viability %). D) Spheroid size changes at day 7 compared to day 0 for all drug treatment groups. Statistically significant differences compared to control condition (0 μm) were *p < 0.05, ** *p* < 0.01, and *** *p* < 0.001. NS is nonsignificant statistically (*p* ≥ 0.05). E) In situ monitoring apoptosis in organoids using Caspase 3/7 green assay. Upper images are green fluorescence signal at 48 h and 72 h. Bottom graph is fluorescence mean intensity over time.

After characterizing organoid cultures in a microfluidic device, we proceeded with testing chemotherapies. Exome sequencing of PDO‐001 revealed the presence of CADPS2‐BRAF chromosomal rearrangement (mutation) (**Table** **1**).^[^ ^28^ ^]^ This mutation upregulates signaling along the RAS‐MEK‐ERK axis, promoting cancer cell proliferation, survival, and transformation.^[^ ^29^ ^]^ Based on these considerations, En and Bi, inhibitors of RAF and MEK respectively, were chosen as targeted therapies for treating PDO‐001. Responses of PDAC organoids to these molecularly targeted therapies were compared to GEM – a front‐line chemotherapy that inhibits DNA synthesis and leads to apoptosis of cancer cells (see **Figure** **5** **A** for chemical structures).^[^ ^30^ ^]^ Five experimental groups were compared: 1) control group without drug, 2) En treatment, 3) Bi treatment, 4) combination treatment of En+Bi, and 5) GEM treatment.

**Table 1:** Clinical information for 3 PDAC patients.

Patient	Molecular profiling	Stage	Grade
PDO‐001	CADPS2‐BRAF chromosomal rearrangement, TP53 (R282W), PTPN13 (4258+2T>C), TMB (2.6), MSS (RNAseq: Overexpressed MET, EZH2, HRAS and NRAS, and Underexpressed CDKN2A)	Stage IV	Moderate to poorly differentiated
PDO‐002	KRAS (G12R), TP53 (R175H), CNL in CDKN2A, CDKN2B, MTAP and SMAD4, TMB (2.6), MSS (RNAseq: MRC2‐PRKCA chromosomal rearrangement)	Stage IV	Moderately differentiated
PDO‐003	KRAS (G12C), ATM (3712_3716del), TMB (4.69)	Stage IV	Moderately differentiated

TMB: Tumor mutational burden

MSS: Microsatellite stable

CNL: Copy number loss

Given the proliferative nature and relative abundance of cells available from PDO‐001, we seeded single cells into the microfluidic device type 1 (see Figure 2). As mentioned previously, single cells were given 2 days to form spheroids in a microfluidic device after which drugs were administered for 7 additional days (see Figure 5A for experimental workflow). Figure 5B shows brightfield and live/dead staining images of microfluidic organoid cultures before (Day 0) and after (Day 7) drug treatment. While organoids without therapy proliferated and retained high viability (≈96%) after 7 days of culture, different treatment groups and drug concentrations resulted in varying levels of cancer toxicity. As quantified in Figure 5C,D, organoids were more sensitive to Bi with ≈55% viability observed for 5 µM Bi, compared to En where loss in viability was minimal (≈90.14%) after exposure to 10 µM En. We next tested whether En and Bi have synergistic effect and found that 2.5 µM En in combination with 2.5 µM Bi had a similar effect on tumor regression as 5 µM Bi alone, but not for higher concentrations (see Figure 5B–D and Figure S10A, Supporting Information). In addition to carrying out Live/Dead staining and monitoring spheroid size change, we also used Caspase‐3/7 apoptosis assay to assess cytotoxic effects of drugs. These results, summarized in Figure 5E and Figure S11 (Supporting Information), support our observations of Bi being a more effective therapy compared to En as well as high sensitivity of these organoids to GEM (Figure S10B, Supporting Information). The results described here illuminate a common clinical scenario where actionable mutations are identified by sequencing but functional responses to these drugs still need to be confirmed experimentally for a given patient. In our case, BRAF‐mutated patient‐specific organoids were significantly more sensitive to MEK inhibitor Bi compared to RAF inhibitor En.

The response of BRAF‐mutated PDO‐001 was compared to BRAF wild‐type PDAC cells, PDO‐002 and PANC‐1 cell line.^[^ ^31^ ^]^ After 7 days of treatment with either En or Bi or the combination of the two drugs, PDO‐002 had only minimal loss in viability at concentrations reaching 10 µM (Figure S12A,B, Supporting Information). PANC‐1 spheroids were similarly unaffected by individual treatment with En or Bi but did exhibit a modest loss in viability (≈10%) and more pronounced decrease in proliferation for combination treatment at concentrations exceeding 2.5 μM (Figure S13, Supporting Information). In summary, we demonstrate that BRAF‐mutant PDAC organoids cultured in a microfluidic device responded to molecularly targeted therapy drugs En and Bi while BRAF‐wild type cells did not. This is another indication that microfluidic organoid cultures allow to accurately assess treatment responses.

### 2.5 Testing Efficacy of a Selective GSK‐3*β* Inhibitor in Combination with GEM

We have previously shown that GSK‐3*β* is overexpressed in PDAC and that its knockdown or pharmacologic inhibition leads to decreased proliferation and induction of apoptosis.^[^ ^32^ ^]^ Furthermore, we used a GSKI, 9‐ING‐41, to demonstrate that GSK‐3*β* plays an essential role in sensitizing PDAC cell lines to GEM.^[^ ^33^ ^]^ As the next step, we wanted to assess the effects of GSKI and GEM using more physiological PDO cultures. We seeded intact organoids into the device type 2 as described in Section 2.2, allowed organoids to acclimate for 2 days and then introduced drugs.

**Figure 6:**
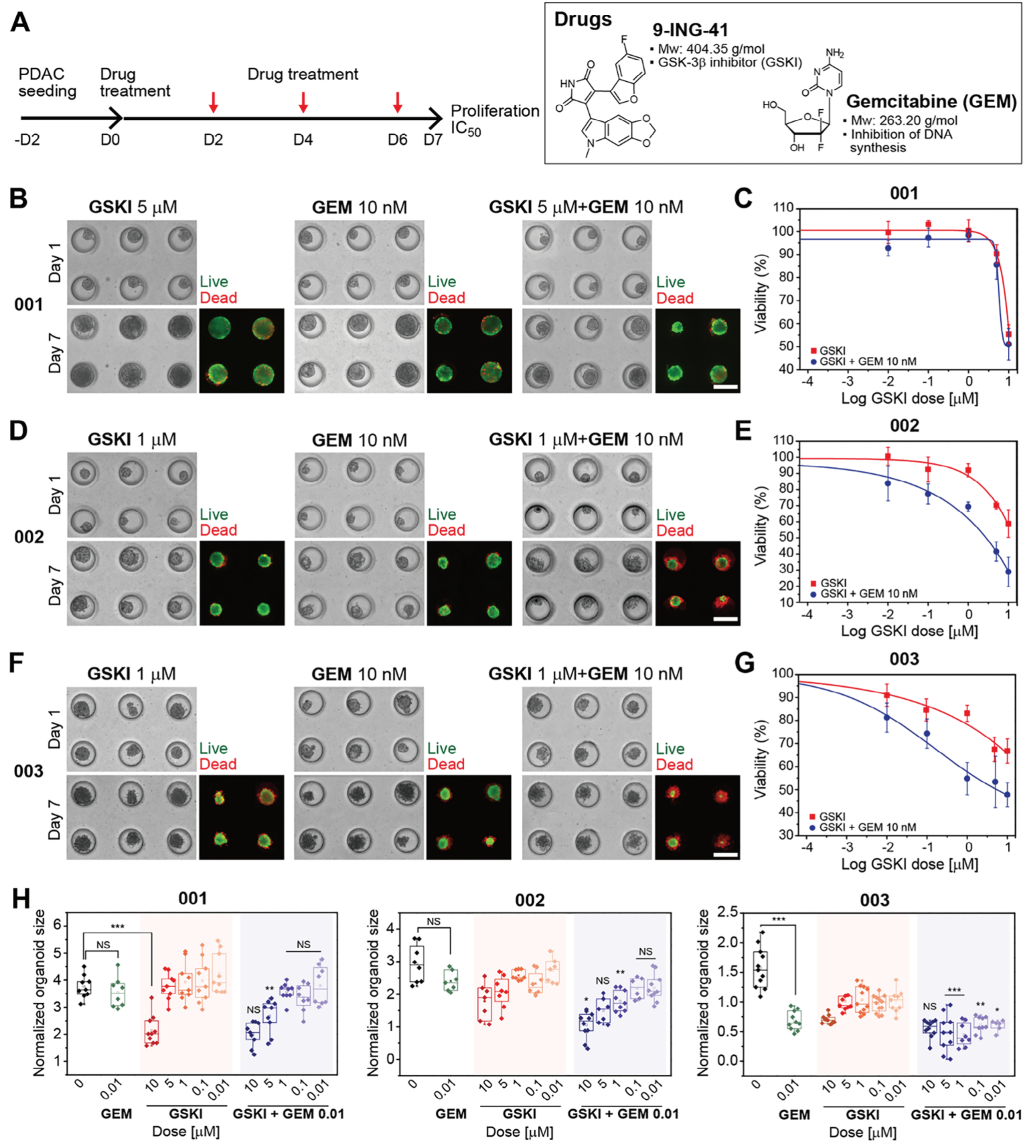
Combination therapy of GSKI and GEM testing using three patient‐specific microfluidic organoid cultures. A) (Left) Experimental timeline for cell seeding and drug treatment. (Right) Chemical structures and molecular weight of 9‐ING‐41 (GSKI) and GEM. B, D, and F) Images of organoids after 1‐day or 7‐day treatment with individual drugs or combination of drugs. Live/Dead staining performed on day 7 to quantify response to therapy. C, E, and G) Relative drug response of single (GSKI) and dual (GSKI+GEM) treatment quantified based on Live/Dead staining at day 7 (Viability %). H) Relative change in organoid size after treatment. Area of organoids at day 7 of treatment was normalized to day 1 (before treatment). Statistical significance of combination treatment (GSKI+GEM) when compared to GSKI treatment alone (NS *p* > 0.05, * *p* < 0.05, ** *p* < 0.01, and *** *p* < 0.001).

The timeline for administering combination chemotherapy is described in **Figure** **6**A. Prior to testing combination therapy, we performed dose‐response study for GEM alone for all three PDOs. As shown in Figure S14 (Supporting Information), the IC50 values were 129 nM, 67 nM, and 500 nM for PDO‐001, ‐002, and ‐003 respectively. These results revealed that GEM caused minimal cytotoxicity at concentrations of 10 nM and lower. We therefore chose to carry out synergy analysis using 10 nM GEM and varying concentrations of GSKI. As may be appreciated from Figure 6B–G, patient‐specific differences in combination treatment were observed. Limited cytotoxicity was observed in PDO‐001 organoids exposed to drugs (Figure 6B,C and Figure S15A, Supporting Information) whereas PDO‐002 and ‐003 did show enhanced response to combination treatment in all concentration ranges (Figure 6D–G and Figure S15B,C, Supporting Information). Synergism scores, which indicate synergistic effect when less than 1, were calculated using Calcusyn software and were determined to be >2.193 for PDO‐001, 0.347 for PDO‐002, and 0.178 for PDO‐003, in all concentrations.^[^ ^33^, ^34^ ^]^

The results described in Figure 6 are exciting for several reasons. First, to the best of our knowledge, this is the first assessment of a novel GSKI using PDOs. Second, responses of organoids parallel previous pre‐clinical and clinical observations suggesting that pre‐treatment with GSK‐3*β* inhibitor sensitizes patients with PDAC to front‐line therapy of GEM.^[^ ^35^ ^]^ Third, we observed patient‐to‐patient variability of organoid responses to combination therapy. Lack of response in PDO‐001 may be attributed to BRAF mutation and MET overexpression which have been reported to decrease sensitivity to GSKI by activating nuclear factor kappa‐B (NF‐κB) pathway downstream of GSK‐3β.^[^ ^29^, ^35^, ^36^ ^]^ A modest synergistic effect in PDO‐002 and ‐003 may be attributed to mutation of KRAS‐G12 (Table 1), which has been shown to increase GSK‐3*β* gene expression and render cancers resistant to GSKI treatment.^[^ ^37^ ^]^ We note that the novel GSKI tested here is not being used clinically and therefore it was not possible to correlate response of microfluidic organoids in patients. However, evidence presented in our study—1) selective response of BRAF mutant PDO‐001 but not BRAF wild‐type PDO‐002 to MEK and RAF inhibitor and 2) difference in responses to GSKI based on molecular profile of each PDO—point to excellent utility of microfluidic organoid cultures for assessing molecularly targeted therapy.

### 2.6 Assessing Combination Cancer Immunotherapy of NK Cells and TriKE Immunomodulators Using Microfluidic Organoid Cultures

Microfluidic devices allow us to position cancer organoids in an array format and maintain their phenotype for at least 9 days while monitoring changes in dimensions of individual organoids. These capabilities make our platform particularly useful for testing immunotherapies by quantifying interactions between immune cells and cancer organoids. Cancer immunotherapy is a novel type of treatment that may entail stimulation of patient's own immune cells to enhance anti‐tumor activity or an adoptive transfer of immune cells.^[^ ^38^ ^]^ While immune checkpoint inhibitor and CAR T‐cell therapies have been used more extensively,^[^ ^39^ ^]^ NK cells are emerging as an exciting alternative immunotherapy strategy because they can attack tumors without prior activation or sensitization.

Unlike T cells that are restricted to recognizing specific human leukocyte antigen (HLA) class I in the context of antigen peptide, NK cells are not HLA restricted. This means that NK cells need not be autologous and are increasingly seen as a potential “off‐the‐shelf” product to be used in the allogenic setting for cancer immunotherapy.^[^ ^40^ ^]^

Anti‐tumor activity of NK cells may be further enhanced by using therapeutics, such as Tri‐specific Killer Engagers (TriKEs).^[^ ^41^ ^]^ TriKEs are engineered immunomodulators that create a synapse between immune cells and cancer cells, mediating tumor suppression.^[^ ^42^ ^]^ Several recent reports highlight the therapeutic potential of TriKEs targeting immune checkpoint B7‐H3 on cancer cells.^[^ ^42^, ^43^ ^]^ Vallera et al. developed a TriKE that is formed by a single domain nanobody specific for B7‐H3 linked to recombinant IL‐15, and a scFv that binds to the activating receptor CD16 on NK cells.^[^ ^42^ ^]^ B7‐H3 (or CD276) is a surface marker expressed in several cancer types including PDAC,^[^ ^43b,c^ ^]^ while CD16 receptor triggers antibody‐directed cell‐mediated cytotoxicity (ADCC) in NK cells.^[^ ^41^, ^44^ ^]^ While described for the first time 2 years ago, anti‐B7‐H3 TriKE has not yet been tested with patient‐derived cancer organoids. Thus, we employed microfluidic cultures of PDAC organoids to test effects of NK cells and anti‐B7‐H3 TriKE.

**Figure 7:**
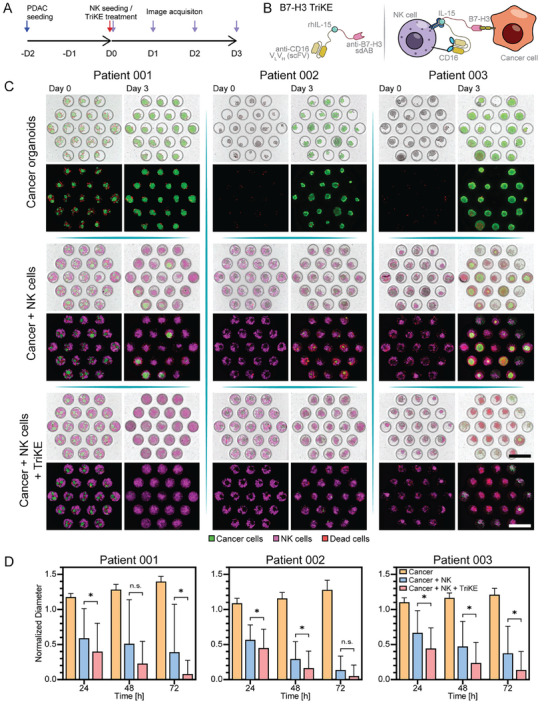
Testing NK cell‐based immunotherapy using microfluidic organoid cultures. A) Timeline and workflow of immunotherapy testing. “D” denotes the day at which each step was completed. B) Schematic describing structure and function of immunomodulator B7‐H3 TriKE. This molecule contains two Ab fragments, anti‐CD16 targeting NK cell marker, and anti‐B7‐H3 specific to immune checkpoint on cancer cells, as well as recombinant IL‐15 to stimulate NK cells. (C) Representative images of microfluidic organoid cultures with and without NK cells/ B7‐H3 TriKEs at day 0 and day 3 of culture. Scale bars = 500 µm. Organoids from three patients were tested. D) Normalized changes in organoid diameter during 3 days of treatment (NS *p* > 0.05 and * *p* < 0.05).

Three treatment groups were compared: (1) a control group consisting of cancer organoids only, (2) a co‐culture group of NK cells and cancer organoids, and (3) a group where cancer organoids were co‐cultured with NK cells in the presence of the anti‐B7‐H3 TriKE. Cancer toxicity in microfluidic devices type 2 (direct organoid seeding device) was monitored over the course of 72 h to assess therapeutic efficacy. The experimental timeline is shown in **Figure** **7** **A** and a schematic of the mechanism by which anti‐B7‐H3 TriKE enhances killing of tumor cells by NK cells is shown in Figure 7B. We supplemented the organoid media with IL‐2 for NK cell maintenance and added SYTOX orange to visualize dead cells.

For all 3 PDOs, the monocultures of cancer organoids showed sustained growth inside microfluidic devices over 3 days of culture (Figure 7C, 72 h). The observed growth was on par with our control experiments during chemotherapy testing, suggesting that addition of IL‐2 and SYTOX orange to the culture media did not adversely affect organoid proliferation. To assess organoid growth in a quantitative manner, we tracked the diameter of individual organoids every 24 h, normalized the diameter at 24, 48, and 72 h to the initial size at 0 h and plotted the average organoid diameter per condition for all PDAC samples (Figure 7D). In the absence of B7‐H3 TriKE, NK cells showed a sustained killing of cancer cells, decreasing the size of the cancer organoids by 35–45% at 24 h, and by 85% at 72 h for PDO‐002, with PDO‐001 and ‐003 decreasing in size by 70% at 72 h. In comparison, the presence of the anti‐B7‐H3 TriKE increased cytotoxicity in all PDOs. All cancer organoids showed a decrease in size of ≈60% at 24 h, a 15–25% improvement compared to non‐TriKE conditions, with a 90–95% size decrease for all PDOs (Figure 7D) at 72 h timepoint. Our results suggest that anti‐B7‐H3 TriKE improved cytolytic activity of NK cells toward cancer organoids. These results also highlight utility of microfluidic cancer organoid cultures for testing immunotherapies, in addition to the chemotherapy studies discussed above.

As noted above, we and others have shown that culturing cells in a microfluidic device in the absence of convection enhances autocrine and paracrine signaling compared to standard (large volume) cultures. We therefore hypothesized that signaling of such inflammatory cytokines as interferon‐gamma (IFN‐γ) may be enhanced in the microfluidic device. To test this hypothesis, we compared PDO‐NK cell co‐cultures in the microfluidic device (type 1) and in the microwell array described in Figure 2F. Immunofluorescence staining for STAT3, an element in the interferon response pathway, revealed higher levels of phosphorylation (pSTAT3) in the microfluidic device compared to microwell array after 2 days of culture (Figure S16, Supporting Information). In addition, more NK cells were observed to infiltrate PDOs in the microfluidic device. These results highlight an additional advantage of microfluidic cultures for testing immunotherapy—amplified autocrine/paracrine inflammatory signaling. Interestingly, the observation of enhanced infiltration of NK cells in the microfluidic device is aligned with sequencing results pointing to the upregulated expression of CCL5, CCL7, and CXCL14—cytokines associated with chemotaxis of NK cells.^[^ ^24^ ^]^

## 3 Conclusion

In this study, we developed and characterized novel microfluidic cultures of PDAC organoids. We demonstrated microfluidic devices to be superior to microwell array format in terms of organoid proliferation and cancer phenotype expression as well as in activation and infiltration of immune cells. RNA sequencing analysis revealed microfluidic organoid cultures to be similar to the gold‐standard Matrigel cultures, with key differences of better oxygenation and priming for chemokine signaling and immune cells infiltration in the microfluidic format. Compared to Matrigel cultures, microfluidic organoids were advantageous for therapy testing because of 1) uniformly sized organoids that may be easily monitored over time to assess response to therapy, 2) the ability to introduce immune cells for testing immunotherapy, and 3) minimal reliance on Matrigel for organoid maintenance. Importantly, microfluidic organoids accurately responded to molecularly targeted therapy. BRAF‐mutant PDOs were shown to be responsive to MEK and RAF inhibitors while BRAF wild‐type PDOs were resistant. Our study also confirmed previous pre‐clinical and clinical observations that GSKI may be used to sensitize tumor to front‐line therapy of GEM. Importantly, we evaluated a novel, recently developed GSKI, 9‐ING‐41, that has not yet been tested with PDOs. In addition to testing chemotherapy or molecularly targeted therapy, our platform is also well‐suited for testing immunotherapy. To demonstrate this point, we assessed a novel immunotherapy comprised of NK cells and an immunomodulatory biologic, TriKE. We demonstrated that cytolytic activity of NK cells may be further enhanced by the presence of TriKE.

In summary, we described a novel microfluidic organoid culture platform for testing patient‐specific responses. This platform helps maintain cancer organoids in a manner similar to gold‐standard Matrigel cultures, enables drug testing with minimal amount of organoids available from a needle biopsy and is well‐suited for testing immunotherapies. In the future, our microfluidic platform may be integrated with biosensing capabilities for quantitative assessment of individual organoids and may be developed into a companion diagnostic for personalized cancer therapy.

## 4 Experimental Section

### Materials

Advanced DMEM/F‐12, DMEM/F‐12 no phenol red, 4‐(2‐hydroxyethyl)−1‐piperazineethanesulfonic acid (HEPES), Glutamax, penicillin/streptomycin (P/S), Fetal bovine serum (FBS), RPMI 1640, LIVE/DEAD viability/cytotoxicity Kit for mammalian cells, Goat anti‐Mouse IgG (H+L) Cross‐Adsorbed Secondary Antibody Alexa Fluor 546, Monoclonal E‐cadherin Mouse Antibody, and CellEvent Caspase‐3/7 Green Detection Reagent were purchased from Thermo Fisher Scientific (Waltham, MA, USA). N2 supplement, B27 supplement, epidermal growth factor (EGF), fibroblast growth factor 10 (FGF10), Cultrex PathClear Reduced Growth Factor BME, Type 2 (Matrigel), and Human Cytokeratin 19 Antibody were obtained from R&D systems (Minneapolis, MN, USA). SB202190 (p38 MAP) and recombinant human IL‐2 were acquired from Peprotech Inc. (Rochy Hill, NJ, USA). N‐acetyl‐L‐cysteine (NAC), Nicotinamide, Gastrin I human, Collagenase type IV, Pluronic F‐127, CryoStor cell cryopreservation media, and Bovine serum albumin (BSA) were purchased from Sigma‐Aldrich (St. Louis, MO, USA). Gentle cell dissociation reagent (GCDR) and Rosette Separation NK cell isolation kit were purchased from STEMCELL Technologies (Vancouver, Canada). Gemcitabine solution (38 mg mL^−1^) was purchased from the Mayo Clinic pharmacy and Y‐27632, Encorafenib, Binimetinib, and 9‐ING‐41 (GSK‐3*β* inhibitor; GSKI) were acquired from MedChemExpress (Monmouth Junction, NJ, USA). Monoclonal Ki‐67 Mouse Antibody, Phalloidin‐iFluor 488, and Phospho‐STAT3 Rabbit Antibody were purchased Cell Signaling Technology (Danvers, MA, USA) and Abcam (Cambridge, UK), respectively. STAT3 Monoclonal antibody was acquired from Proteintech (Rosemont, IL, USA). The PANC‐1 and L‐WRN cell lines were purchased from the ATCC (Manassas, VA, USA).

For PDAC organoid media, conditioned media from L‐WRN is prepared. L‐WRN conditioned media (Wnt‐3A, R‐spondin 3, and noggin conditioned medium) was collected by following previously reported protocol^[^ ^10^, ^45^ ^]^ and stored at −30 °C prior to the preparation of organoid media. The organoid media for organoid culture was based on 50% Advance DMEM/F‐12 and 50% L‐WRN conditioned media, supplemented with 1× HEPES, 1× Glutamax, 1× N2 supplement, 1× B27 supplement, 500 ng mL^−1^ EGF, 1 µg mL^−1^ FGF10, 3 µM SB202190, 0.5 µM A83‐01, 1 mM NAC, 10.25 mM Nicotinamide, 10 nM Gastrin, 1× P/S (100 U mL^−1^ penicillin and 100 µg mL^−1^ streptomycin), and 10 µM Y‐27632.

### Collection of PDAC Biopsies

The patient PDAC biopsy collection was approved by the Mayo Clinic under IRB #17‐003174 and were carried out with the full, informed consent of the subjects. The samples were collected by ultrasound‐guided biopsies using an 18‐gauge needle and were ≈1 mm thick × 10–20 mm long. The procedure was performed at the Mayo Clinic in Rochester, MN. Two to four needle biopsies were made available for research from each biopsy collection. The needle biopsies collected from three different patients were shown in Figure S2 (Supporting Information).

### Organoid Formation and Culture in Matrigel Domes

Needle core biopsies were placed in the tissue storage solution on ice in the procedure room and delivered to the research lab within 30 min of collection. The workflow from biopsy digestion to seeding steps is illustrated in Figure S1 (Supporting Information). Fresh biopsies were placed on a 6 mm‐culture dish with Krebs‐Ringer Bicarbonate (KRB) buffer on ice and minced to fragments of <1 mm in length and width using a sterile disposable blade. The fragments were digested with 2.5 mg mL^−1^ collagenase type IV under mild agitation in a water bath (37 °C) for 5 min. The dissociated supernatant was filtered through a 100 µm cell strainer to remove ECM, and then centrifuged at 1500 rpm for 5 min. To remove red blood cells (RBCs), the dissociated cells were immersed in RBC lysis buffer for 3 min at 25 °C. The effect of the lysis buffer was stopped by adding 10% FBS‐containing PBS. Dissociated cells were then seeded onto Matrigel domes in a 12‐well plate using organoid media. For passaging organoids, Matrigel domes were broken by treating them with GCDR for 1 min, followed by organoids collection. The organoids were resuspended in 50% Matrigel/50% organoid media and dispersed onto a 12‐well plate to make Matrigel domes. After gelation of the Matrigel domes, organoid media was added carefully. The media was changed every 3–5 days. The organoids from patient 1 (PDO‐001) were passaged 10–20 times while PDO‐002 and ‐003 were passaged five to seven times due to the reduced proliferation by successive passages of PDO‐002 and ‐003 after passage No. 8. For later use, the PDOs were cryopreserved using CryoStor following manufacturer's instructions.

### Fabrication of Microfluidic Devices

Two types of microfluidic devices (see Figures 2A and 4A) were fabricated using standard soft lithography techniques.^[^ ^17^, ^46^ ^]^ Device 1 was designed to organize single cells into organoids whereas device 2 was used to seed intact organoids. The detailed protocol for fabricating molds and PDMS devices is described in the Supplementary Information.

Before use, microfluidic devices were degassed in a vacuum desiccator and treated overnight with 1% Pluronic F‐127 in 1× PBS. This treatment minimizes cell attachment to the surface of PDMS and promotes cell aggregation into spheroids.^[^ ^47^ ^]^ The devices were then washed with 1× PBS to remove the Pluronic solution and exposed to UV light for 30 min for sterilization. Prior to cell seeding, PBS solution was removed from media reservoirs.

### Assessing Interactions of Chemotherapy Drugs with PDMS‐Based Microfluidic Devices

The concentrations of each drug (En, Bi, GSKI, and GEM) after incubation in microfluidic device were quantified by UV–Vis spectroscope (NanoDrop One‐C; Thermo Fisher Scientific). Four different drugs were dissolved in 10% FBS‐containing phenol red‐free DMEM/F‐12 media at 10, 50, and 100 μM, excepting for 100 mM GSKI due to the severe sedimentation during incubation. Then, the drug solutions were incubated in the microfluidic devices and stored at 37 °C incubator for 2 days. The solution was recirculated every day. To mimic chemotherapy testing with PDOs, the device was pretreated with Pluronic F‐127 for 1 day and 10% FBS‐containing phenol red‐free DMEM/F‐12 media for 2 days before adding drugs. After 2 days of incubation, the drug solutions were collected and analyzed by UV–Vis spectroscopy. Calibration curves were created by measuring absorbance values for drug concentrations ranging from 0 to 1250 μM, unknown samples from drugs after incubation were then determined using these calibration curves.

### Characterization of PDAC Organoids by Immunofluorescence and RT‐PCR

Size (area) changes of PDAC organoids in Matrigel or wells were measured using ImageJ software. For immunostaining, whole PDAC organoids were fixed and permeabilized with 4% Paraformaldehyde (PFA; Electron Microscopy Sciences) and 0.1% Triton X‐100 (Sigma‐Aldrich) for 30 min, respectively, and blocked with 2% BSA for 1.5 h at 25 °C. Then, the primary antibodies and fluorophore‐conjugated secondary antibodies with DAPI (BD Biosciences) and Phalloidin‐Alexa 488 in 1% BSA solution were sequentially treated for 1–1.5 h, at 25 °C. For staining in microwells, 200 µL of each solution was dispensed into the media reservoirs and recirculated every 15 min. the organoids were imaged using confocal microscopy (LSM 780; Carl Zeiss, Germany).

For RT‐PCR analysis, PDO‐001 was cultured in microwells format and microfluidic device for 5 days. Then, total RNA was isolated using a commercial kit (RNeasy kit, Qiagen (Valencia, CA, USA)) following the manufacture's protocol. Approximately 20–30 ng of total extracted RNA was used for synthesis of cDNA, following the reverse transcription kit instructions (Roche). The primer sequences used for RT‐PCR were listed in Table S2 (Supporting Information). Gene expression was tested using the QuantStudio 5 System (Thermofisher Scientific) with SYBR Green qPCR Master Mix and was normalized to glyceraldehyde 3‐phosphate dehydrogenase (GAPDH). The amplification procedure for Real‐time PCR consists of 40 cycles of denaturation at 95 °C for 5 s, annealing at 55 °C for 15 s, and extension at 69 °C for 20 s. The final evaluation was carried out based on the threshold cycles using the ΔΔCT method and normalized to expression level of PDAC organoids in Matrigel.

### Preparation of PDAC Organoids for Seeding into Microfluidic Devices

Organoids embedded in Matrigel were expanded for 1–1.5 weeks. Then Matrigel domes were dissociated by treatment with GCDR and gentle pipetting. This digest was centrifuged at 1500 rpm for 4 min at 4 °C conditions to remove Matrigel debris and washed with cold DMEM for removing residual Matrigel thoroughly. At this point in the protocol, we produced intact organoids. These organoids were either seeded intact into a microfluidic device 2 or were broken down into single cells. When dissociating organoids into single cells, digest was exposed to 2 mL of 0.25% Trypsin‐EDTA (Gibco) for 4 min at 37 °C (in water bath) under mild agitation after which DMEM with 10% FBS was added to stop digestion. Clumps of cells or debris were filtered using 100 µm strainer prior to seeding.

### Forming and Maintaining Organoids in Microfluidic Devices

When seeding into the microfluidic device 1, single cell digest was resuspended to a concentration of 4 × 10^6^ cell·mL^−1^ in organoid media with 1% DNase (see Section 4.1. for media description). Then, 100 µL of cell suspension was placed in one of the media reservoirs to create a difference in hydrostatic pressure and drive cells into the device. Positive pressure was applied to one of the reservoirs, by covering the top with a finger and pushing gently, to increase the flow inside the chamber and equilibrating media levels between the cylinders. At this point, the flow in the device stops and cells settle down into microwells. The microfluidic devices were washed twice with fresh media to remove cells that did not land in microwells. Organoid media with 1% Matrigel was added, and devices were moved to an incubator (at 37 °C, 5% CO_2_). The media was recirculated after 24 h of culture by aspirating 50 µL from one media reservoir and dispensing it into another reservoir. Organoid formation was typically observed after 48 h. From this point onwards, organoids were cultured in media without Matrigel.

The microfluidic device type 2 contained a side injection port protected by the normally closed valve to allow opening the port, introduce intact organoids directly into the culture chamber (see Figure 4A), then reseal the port and use the device as a standard petri dish without connection lines. Operation of a device to open the valve was described recently.^[^ ^46^ ^]^ Briefly, a device was connected to a vacuum line and placed on a microscope in a biosafety hood. The valve was opened by negative pressure to allow insertion of 27‐gauge needle and transfer of organoids into the culture chamber containing in microwells. Each device was typically populated with 13 organoids. These organoids settled down into microwells after 20 s under static conditions. Once organoids were confined to microwells, the vacuum line was disconnected, and valve/injection port closed. No external sources of pressure were needed to keep this normally closed valve sealed during a multi‐day experiment. Microfluidic organoids were cultured in organoid media supplemented with 1% Matrigel for the first 48 h. No Matrigel was used in media thereafter.

### Bulk RNA Sequencing

Organoids were cultured in Matrigel as described in Section 4.3 and seeded into microfluidic devices as described in Section 4.7. Three sample replicates from one patient for each type of organoid culture platform (Device and Matrigel) were processed. Culture media was removed and organoids were washed with PBS. Cells were lysed and homogenized for RNA extraction following the Qiazol‐based miRNeasy Micro Kit (Qiagen). Isolated RNA was assessed for quality using RNA fragment analysis and all samples with RNA Integrity Scores (RIN) >7 were processed. RNA samples were cleaned up to ensure any contaminating salts and solvents were removed. A standard input stranded mRNA library prepration was used to focus on the poly‐adenylated transcripts. Subsequently, bulk RNA sequencing data was obtained using paired‐end next‐generation sequencing IlluminaNovaSeqSP (100 cycles).

### Differential Gene Expression Analysis

RNA‐seq FASTQ file pairs were trimmed using TrimGalore version 0.6.4 and Cutadapt version 4.4. FASTQC files from trimming were used to assess RNA quality. Subsequently, trimmed FASTQ files were aligned using STAR version 2.7.1a to the human reference genome GRCh38. Counts for each gene were obtained from STAR output and differential gene expression analysis was performed using the EdgeR version 3.40.2 R package. EdgeR allowed for assessment of similarity between samples using hierarchical clustering of sample distances and principal component analysis to determine if the Matrigel and Device groups had significantly different overall gene expression. Heatmaps were created using the R package pheatmap version 1.0.12 and the volcano plot was generated using ggplot2 version 3.4.3. Genes with a log_2_(fold change) >2 or <−2, and an adjusted *p*‐value of <0.05 were considered significantly differentially expressed.

### Pathway Enrichment Analysis and Gene Set Enrichment Analysis

The R package ClusterProfiler version 4.6.2 was used to identify Gene Ontology (GO) biological processes enriched in the significantly upregulated and downregulated genes. Gene set enrichment analysis (GSEA version 4.3.2) was performed with default settings, specifically, 1000 permutations using the c2.all.v2023.1.Hs.symbols.gmt gene set database and the Human_Ensembl_Gene_ID_MSigDB.v2023.1.Hs.chip Chip platform (http://software.broadinstitute.org/gsea/index.jsp). Gene sets for NOTCH (KEGG_NOTCH_SIGNALING_PATHWAY M7946), WNT (KEGG_WNT_SIGNALING_PATHWAY M19428), and TGF BETA (KEGG_TGF_BETA_SIGNALING_PATHWAY M2642) were obtained from the GSEA Molecular Signatures Database.

### Additional Pancreatic Cancer Datasets

Publicly available Bulk RNA sequencing data of patient‐derived organoids derived from Pancreatic Ductal Adenocarcinoma were retrieved from the NCBI SRA Run Selector under BioProject accession number PRJNA873279 (https://www.ncbi. nlm.nih.gov/geo/). Fastq files were trimmed, aligned, and differentially expressed genes were analyzed as described in Section 4.10.

### Administering Chemotherapy to Microfluidic Organoid Cultures

Chemotherapy testing was carried out in the following manner. First, all the drugs (with the exception of Gemcitabine) were dissolved in DMSO to create 10 mM drug stock solution. Then, chemotherapy drugs were dissolved in organoid media at concentrations ranging from 0.1 nM to 10 µM. DMSO concentration ranged from 10^−7^ to 0.1% for this concentration range. Previous studies have shown that DMSO is nontoxic when used at concentrations below 0.5%.^[^ ^48^ ^]^

The same concentration was used for three treatment arms: 1) Encorafenib (En), 2) Binimetinib (Bi), and 3) Gemcitabine (GEM). Media with drug was added at the 48 h time point once organoids had formed and/or have acclimated to culture conditions. Drug treatment lasted for 7 days with fresh media (containing drugs) changed every 48 h. Media in the devices was recirculated using the method described above where 50 µL from one media reservoir was collected and placed into the other reservoir. Images of microfluidic organoid cultures were acquired every 2 days using an inverted fluorescence microscope (BZ‐X800, Keyence). On day 7 of treatment, the media was removed, and organoids were stained using a Live/Dead staining kit following the manufacture's protocol. The viability was calculated following the equation: (Viability% = 100 × A_green_ / (A_green_ + A_red_)) with area measurements performed using ImageJ software.

For apoptosis assay, 2 µM of Caspase‐3/7 reagent solution in organoid media was applied to each group after 2 days of seeding. The media with reagent was recirculated every day and changed every 2 days. Apoptotic cells emitted green fluorescence and were imaged using a fluorescence microscope (BZ‐X800, Keyence). The fluorescence intensity was calculated using the ImageJ software.

When testing a combination of GSKI and GEM therapy, different concentrations of individual drugs or a combination of drugs were dissolved in organoid media and added to each microfluidic device at day 2 after seeding. Cultivation of organoids and assessment of drug response was carried out in the same manner as described in the previous paragraph.

### PANC‐1 Cell Culture and BRAF‐Targeted Chemotherapy Testing in the Microfluidic Device

The PANC‐1 cells were cultured at 37 °C incubator in 75 T flask until the confluency reaches ≈95%. The medium for PANC‐1 is 90% high glucose DMEM, 10% FBS, and 1% P/S. The culture media was changed every 2–3 days. To perform BRAF‐targeted chemotherapy testing, the PANC‐1 cells were washed with 1x PBS and treated TripLE at incubator for 3 min to detach cells from the flask. Sequentially, cells were collected by centrifugation at 1500 rpm for 4 min and seeded them into the microfluidic device type 1 (single cell seeding device). The process for seeding cells and chemotherapy testing followed previously described in Sections 4.8 and 4.13.

### Isolation and Expansion of NK Cells

Primary human NK cells were isolated from apheresis cones from anonymized healthy donors obtained from the Mayo Clinic Blood Bank (Rochester, MN, USA) as previously described.^[^ ^49^ ^]^ Briefly, human peripheral blood mononuclear cells (PBMCs) were isolated by Ficoll‐Hypaque (GE Healthcare, Chicago, IL, USA) density gradient centrifugation. The resulting PBMCs were then mixed with RBCs at a ratio of 1:100 and incubated with antibodies from Rosette Separation NK cell isolation kit for 20 min at room temperature as per the manufacturer instructions. Ficoll‐Hypaque density gradient centrifugation was then repeated, and the resulting NK cells were analyzed for purity by flow cytometry using anti‐CD3 and anti‐CD56 (BioLegend, San Diego, CA, USA) and Ghost Dye (Tonbo, San Diego, CA, USA).

Primary human NK cells were expanded using the modified mbIL‐21 K562 line, CSTX002, which was kindly supplied by Dean Lee (Ohio State University). Expansion was accomplished as described in Somanchi et al.^[^ ^44^ ^]^ Briefly, mbIL‐21 K562 cells were irradiated at 100 Gy and mixed with NK cells at a E:T ratio of 1:2 for 7 days with fresh medium and IL‐2 supplied on days 3, 5, and 7. This cycle of expansion was repeated three times for a total of 21 days. NK cells were expanded and maintained in RPMI 1640 supplemented with 50 U mL^−1^ recombinant human IL‐2, 10% FBS, and 1% each of Pen Strep, sodium pyruvate, MEM nonessential amino acids, and L‐glutamine from Corning (Corning, NY, USA).

The Tri‐Killer Engager (TriKE) molecule contained an anti‐CD16 antibody fragment, an IL‐15 moiety, and an anti‐B7‐H3 single‐chain variable fragment (scFv). Detailed description of this biologic is provided in a recent paper.^[^ ^42^ ^]^

### Testing NK Cell‐Based Immunotherapy Using Microfluidic Organoid Culture

Organoids were seeded into microfluidic devices as described in Section 4.7. Three experimental conditions were tested: (1) cancer organoids, (2) cancer organoids + NK cells, and (3) cancer organoids + NK cells + TriKE treatment. When working with experimental groups 2 and 3 using NK cells, the following protocol was followed. PDAC cells were given ≈48 h in microfluidic devices for organoid formation before adding NK cells. Immune cells were labeled with Vibrant DiD cell tracker dye and resuspended at a density of 10×10^6^ cell mL^−1^. A 100 µL of cell suspension was infused into each microfluidic device. Excess cells were washed out, and all devices were filled with fresh organoid media supplemented with 50 U mL^−1^ recombinant human IL‐2, and SYTOX orange at a concentration of 5 µM. For experimental group 3, media was supplemented with B7‐H3 TriKE at 3 nM. This concentration was shown to be effective previously. Three microfluidic devices were used for each experimental group. The media was recirculated every 24 h by gently collecting contents of one media reservoir with a pipette and placing these contents into the opposite reservoir. Images of the organoids in microwells were acquired every 24 h using an inverted fluorescence microscope. The protocol described above was used for all three patient samples/PDOs.

### Analysis of Cancer Organoid Killing by NK Cells and STAT3 Activation in NK Cells

For immunotherapy studies, images were acquired with a fluorescence microscope using a 10× objective. A z‐stack was acquired for each microfluidic chamber, obtaining up to 15 slices with a 10 µm pitch, covering all 19 microwells in every chamber. Images were acquired in brightfield, GFP channel for GFP‐transfected PDAC (PDO‐001), TRITC channel for SYTOX orange staining of dead cells, and Cy5 channel for DiD labeling of NK cells. For non‐GFP‐expressing cancer cells, Calcein‐AM (viability marker) was added at the end of the experiment (72 h) to help visualize live organoids. All the images acquired for each microfluidic chamber were processed to obtain a single full‐focus image per channel, by using the microscope software.

Image analysis was done using Fiji software.^[^ ^50^ ^]^ The area for each organoid in all images was tracked over time for every tested condition and PDAC sample. The diameter of each organoid at 24, 48, and 72 h were normalized against 0 h to determine organoid growth or shrinking. For immunofluorescence staining of STAT3 and p‐STAT3, NK cells and PDO‐001 were co‐cultured in microfluidic device (single cell seeding device) and PDMS microwell for 1–3 days and stained for expression of STAT3 and p‐STAT3 as described above. ImageJ was used to quantify fluorescence due to p‐STAT3 and STAT3 staining.

### Statistical Analysis

*T‐test* was performed for all data sets to determine significance between conditions for immunotherapy data. The significance was denoted as *, and NS, which means *p* < 0.05, and nonsignificant (*p* > 0.05), respectively. All the organoids in the three chambers per condition were averaged (*n* ≥ 40). Error bars denote standard deviation. One‐way ANOVA analysis followed by Tukey's post hoc was used to analyze significance when comparing more than two groups. Origin and GraphPad Prism software were used to plot and statistically analyze the data.

## Conflict of Interest

The authors declare no conflict of interest.

## Supporting information

Supporting Information

Supplemental Table 1

## Data Availability

The data that support the findings of this study are available from the corresponding author upon reasonable request.
