# Supplementary material for: Microfluidic Organoid Cultures Derived from Pancreatic Cancer Biopsies for Personalized Testing of Chemotherapy and Immunotherapy
Source: Adv Sci (Weinh). 2023 Nov 29;11(5):2303088. doi: 10.1002/advs.202303088 (PMC10837378; doi:10.1002/advs.202303088)
Supplement: Supplementary file 1 — Supporting Information [file ADVS-11-2303088-s001.pdf]

ADVANCED SCIENCE
Open Access

Supporting Information

for *Adv. Sci.*, DOI 10.1002/advs.202303088

Microfluidic Organoid Cultures Derived from Pancreatic Cancer Biopsies for Personalized Testing of Chemotherapy and Immunotherapy

*Daheui Choi, Alan M. Gonzalez-Suarez, Mihai G. Dumbrava, Michael Medlyn, Jose M. de Hoyos-Vega, Frank Cichocki, Jeffrey S. Miller, Li Ding, Mojun Zhu, Gulnaz Stybayeva, Alexandre Gaspar-Maia, Daniel D. Billadeau, Wen Wee Ma and Alexander Revzin**

Supporting Information

Microfluidic organoid cultures derived from pancreatic cancer biopsies for personalized testing of chemotherapy and immunotherapy

*Daheui Choi, Alan M. Gonzalez-Suarez, Mihai G. Dumbrava, Michael Medlyn, Jose M. de Hoyos-Vega, Frank Cichocki, Jeffrey S. Miller, Li Ding, Mojun Zhu, Gulnaz Stybayeva, Alexandre Gaspar-Maia, Daniel D. Billadeau, Wen Wee Ma and Alexander Revzin**

Supplementary information for fabrication of devices

The two types of microfluidic devices used in this work (see **Figure 2A and 4A**) were fabricated using soft lithography techniques.[1] Device type 1 was fabricated using two SU-8 molds, one for the microwells layer and another for the flow layer. Device type 2 was fabricated using three SU-8 molds: 1) microwell layer, 2) flow layer and 3) valve layer. Both microfluidic devices were designed using AutoCAD 2019 (Autodesk). The flow layer mold for device 1 had a height of 100 µm, while all the other molds had a height of 300 µm. To fabricate the mold with a 100 µm thickness layer, SU-8 2050 (Kayaku Advanced Materials) resist was spin coated on a pristine 4-inch silicon wafer at 1,400 rpm. The wafer was soft baked following the manufacturer recommendations. Afterwards, the device design was exposed on the resist using a micropattern generator (µPG 101, Heidelberg). After post exposure bake, the wafer was developed for ~5 min in developer solution (SU-8 Developer, Kayaku Advanced Materials) and then hard baked at 160°C for 15 min. As the last step, the wafer was exposed to chlorotrimethylsilane on a closed chamber for at least 30 min. The four remaining molds were fabricated in a similar fashion, with the only difference that SU-8 2100 was spin-coated at 900 rpm to create a 300 µm resist layer. All molds were placed on a 150 mm petri dish.

The microfluidic devices were fabricated by replica molding using polydimethylsiloxane (PDMS, Sylgard 184 elastomer kit, DOW). For device 1, the PDMS base and curing agent were mixed in a 10:1 ratio and poured on the molds. The molds were then baked for 60 min at 80°C, the devices was cut and PDMS peeled off from the molds. Inlets and outlets of the microfluidic chambers were punched using 14-gauge syringe needles. The devices were oxygen plasma treated for 20 s in a plasma asher (YES) and then assembled. Cloning glass

cylinders (outer diameter of 8 mm) were bonded at the inlet/outlets of the device to create media reservoirs.

For microfluidic device 2 (containing injection port), 10:1 PDMS mix was poured into the microwell layer mold to reach a height of ~1 mm and baked for 60 min at 80°C. The PDMS was then cut and peeled off from the mold and set aside. A PDMS mix at 20:1 ratio was poured into the flow layer mold to reach a height of ~0.5 mm, and a PDMS mix at 5:1 poured into the valve layer mold to a height of 4-5 mm. The molds were baked for 20 min at 80°C. The PDMS from the valves mold was cut, devices peeled off, holes punched, and then aligned on top of the flow layer mold using a stereoscope. The chambers mold was baked for 90 min at 80°C to bond the PDMS layers. Each microfluidic device was cut and peeled off from the mold. Inlets and outlets from the chambers were punched and the two layers were bonded to the microwells PDMS slab by oxygen plasma treatment. The injection port area was protected from the plasma treatment by placing a strip of invisible tape (2 × 7 mm) of both PDMS layers to prevent bonding in that area. Glass cylinders were attached to the inlets and outlets of the devices.

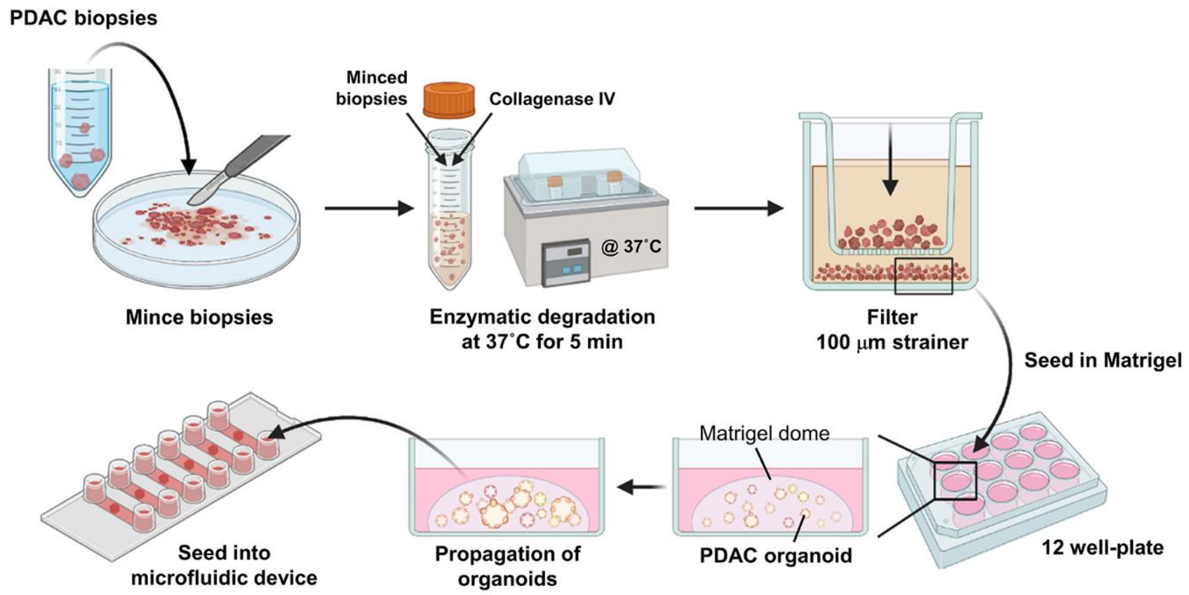

Figure S1. Schematic illustration of PDAC organoid formation from biopsies. Biopsy cores were minced and digested using collagenase IV. Cell digest then passed through a 100 μm strainer. Cells were seeded into 12-well plates in Matrigel domes and cultured in media promoting organoid formation. One or two weeks after seeding, organoids were passaged into additional wells. After sufficient amount of material was generated, organoids were either placed into microfluidic devices or cryopreserved for future use.

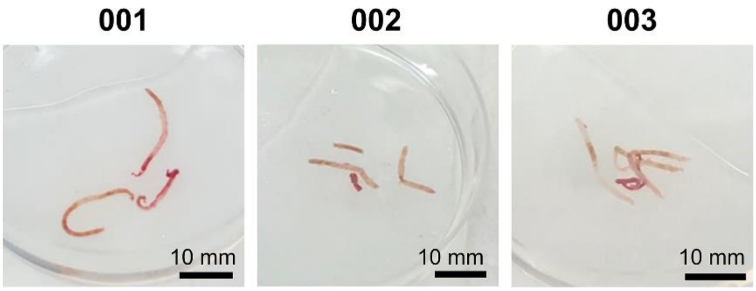

Figure S2. Needle core biopsies from 3 pancreatic cancer patients.

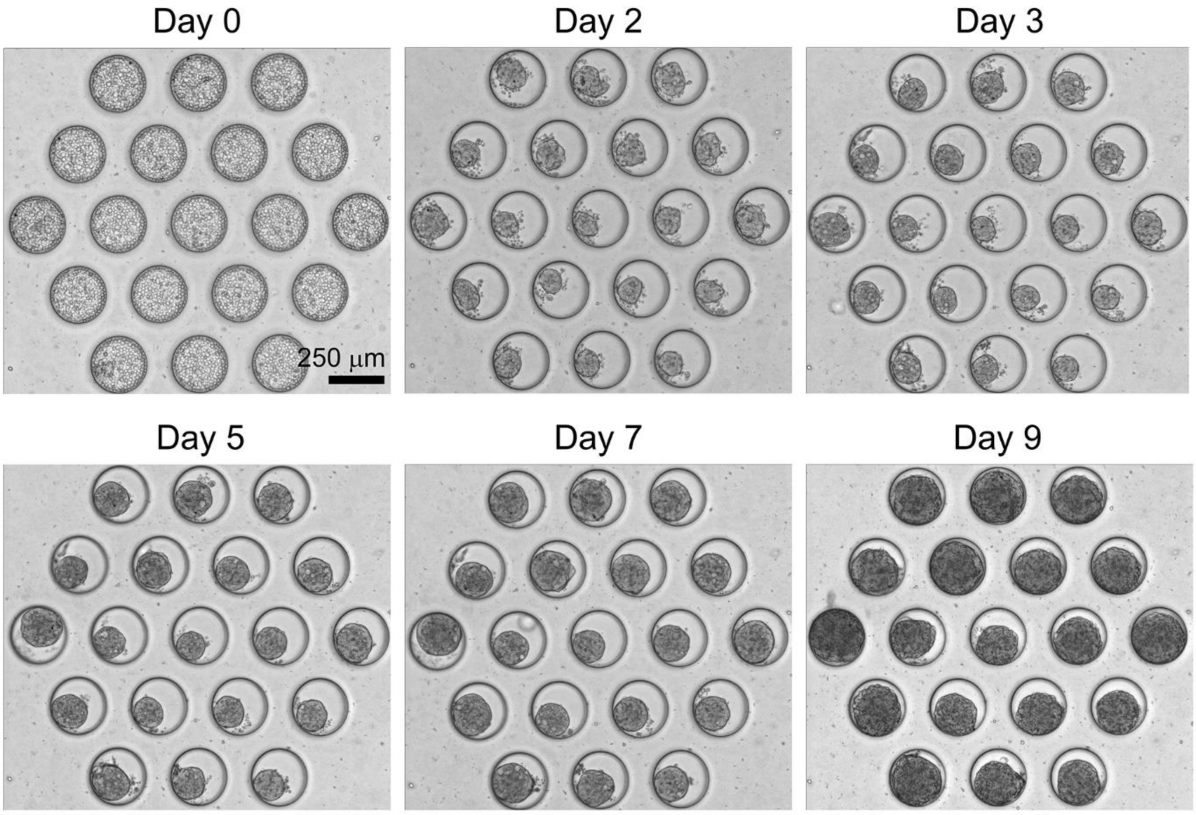

Figure S3. Images of organoid formation and growth in a microfluidic device type 1.

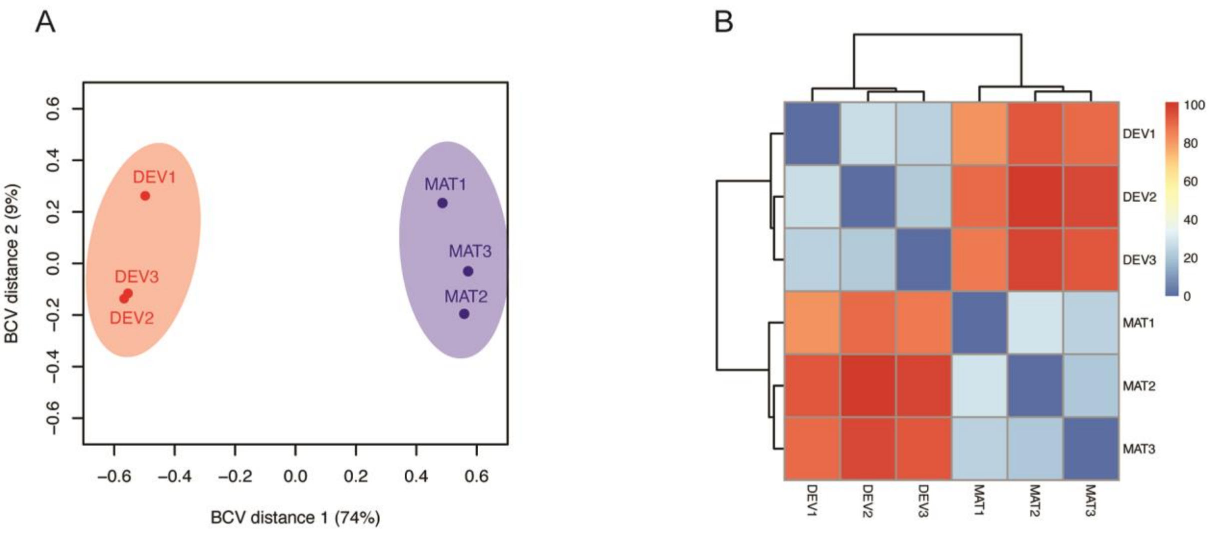

C

Highly Enriched in Matrigel

Symbol	P-Value	logFC	Neglog10P-Value
ANKRD1	2.10E-24	-5.4515669	22.7724659
SLC2A3	6.66E-216	-5.2870199	212.31253
FGF8	5.82E-10	-5.2459448	8.71601706
H19	1.05E-39	-5.2055536	37.7485916
KRT4	2.03E-26	-5.1820883	24.7371357
KISS1	5.13E-16	-5.0942902	14.5886248
ANGPTL4	0	-4.9569978	Inf
DPPA2	1.50E-08	-4.9510976	7.35422186
KRT13	4.03E-116	-4.570289	113.184083
RBP3	1.75E-34	-4.5405738	32.6246799

D

Highly Enriched in Devices

Symbol	P-Value	logFC	Neglog10P-Value
GLYATL2	7.23E-20	8.39468161	18.3410939
PALMD	1.83E-21	8.35296315	19.9008659
ESM1	1.50E-73	7.49105746	71.1012558
CXCL13	5.42E-10	6.97670305	8.74585126
IRS4	2.82E-10	6.93958162	9.02056789
PI3	0	6.5774319	Inf
PLEKHS1	2.61E-53	6.56915873	51.1252754
KRT6B	2.59E-56	5.54474508	54.0847981
RP6-24A23.7	4.18E-12	5.4219475	10.7930205
C4orf40	4.74E-11	5.3158457	9.7691021

Figure S4. Transcriptomic comparison of microfluidic device and Matrigel-based organoid cultures. (A) MDS Plot between PDOs in microfluidic compared to Matrigel culture. Samples are plotted on a two-dimensional scatterplot such that distances on the plot approximate the expression differences between the samples. 500 top expressed genes from each sample were used to calculate pairwise distances between samples. (B) Heatmap of

sample to-sample distances between microfluidic culture compared to Matrigel culture PDO replicates (C) Top 10 differentially downregulated genes in between PDOs in μFD compared to Matrigel culture. (D) Top 10 differentially upregulated genes in between PDOs in μFD compared to Matrigel culture.

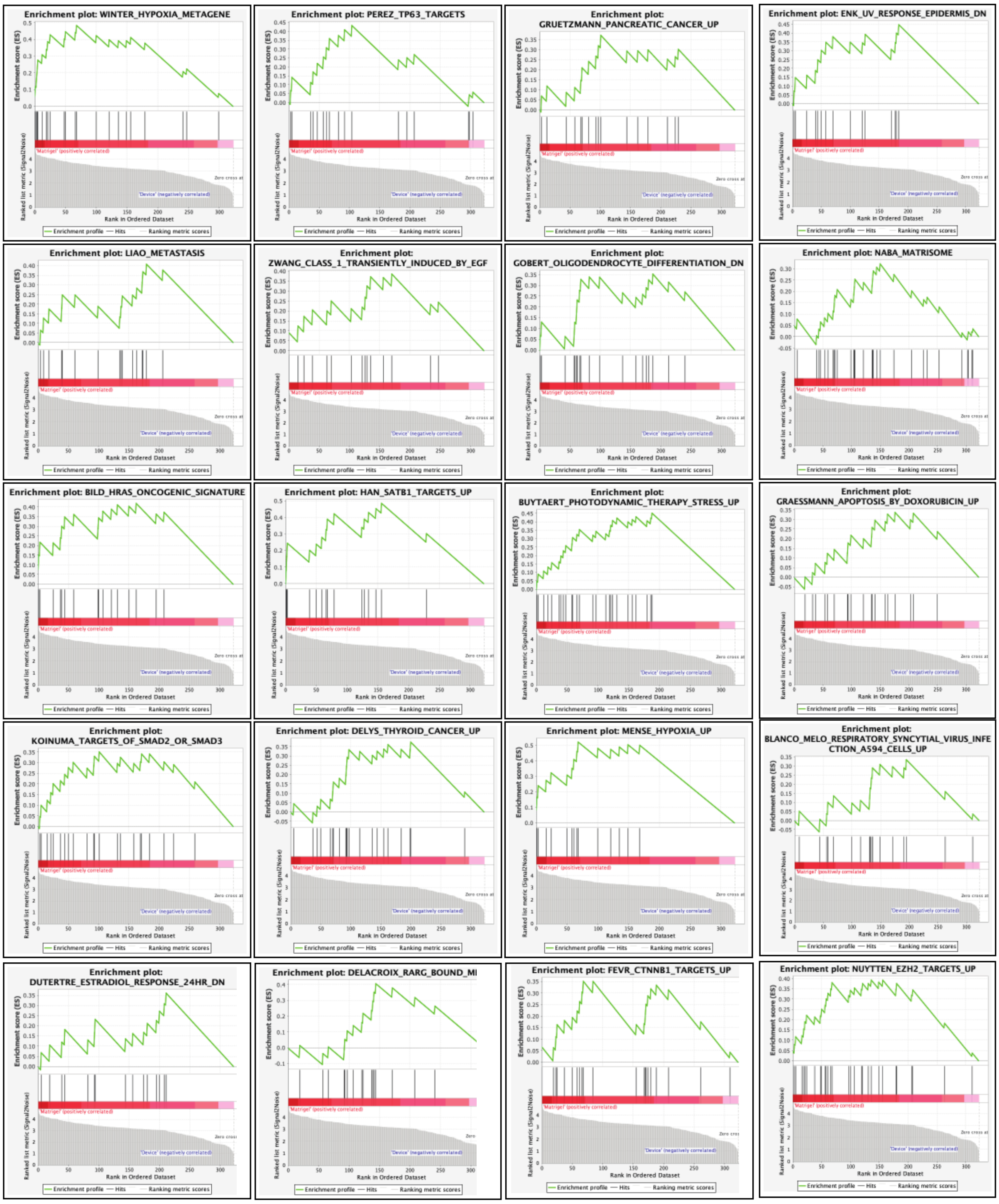

Figure S5. Top 20 GSEA enrichment plots highlighting key pathways enriched in Matrigel culture.

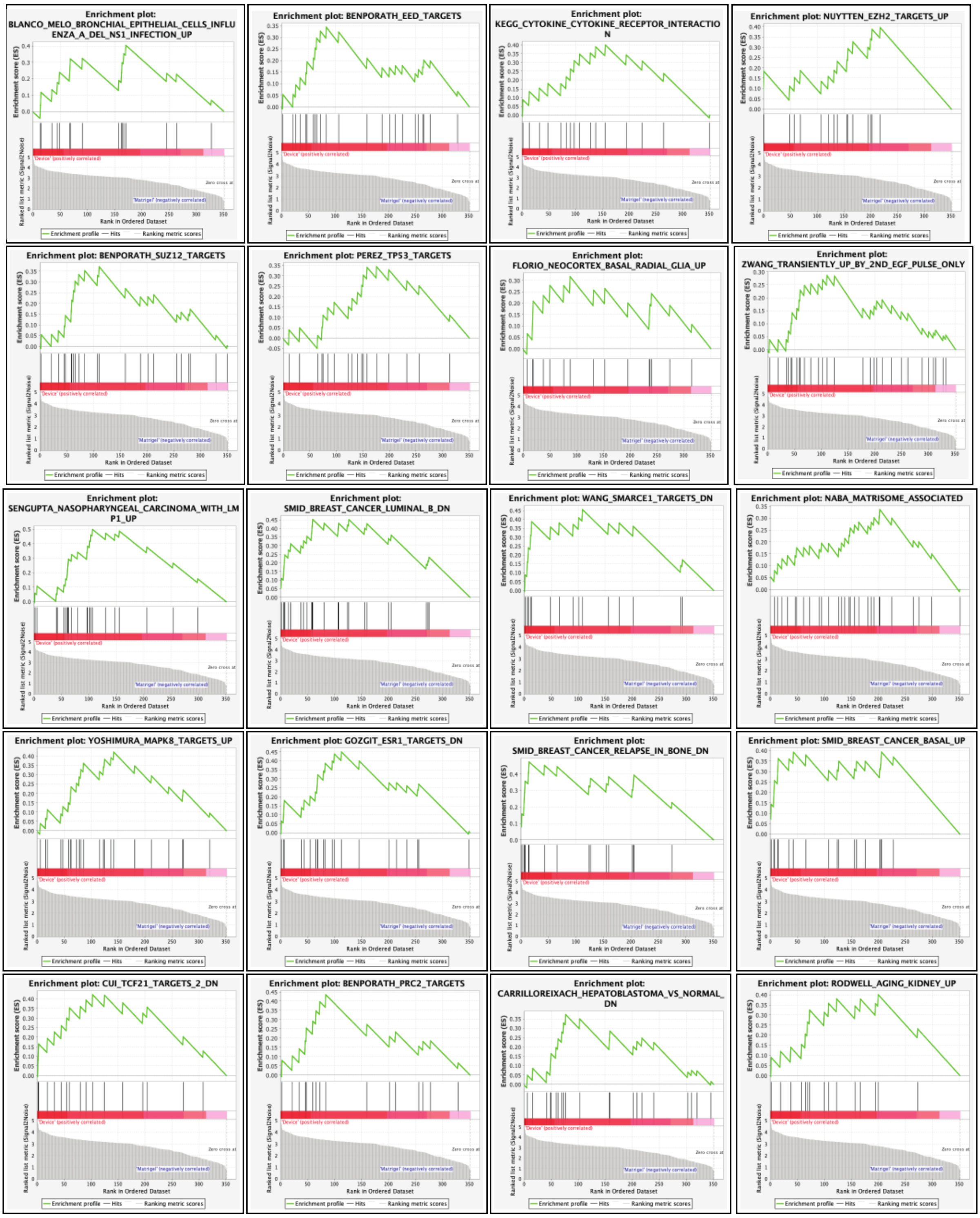

Figure S6. Top 20 GSEA enrichment plots highlighting key pathways enriched in Microfluidic culture.

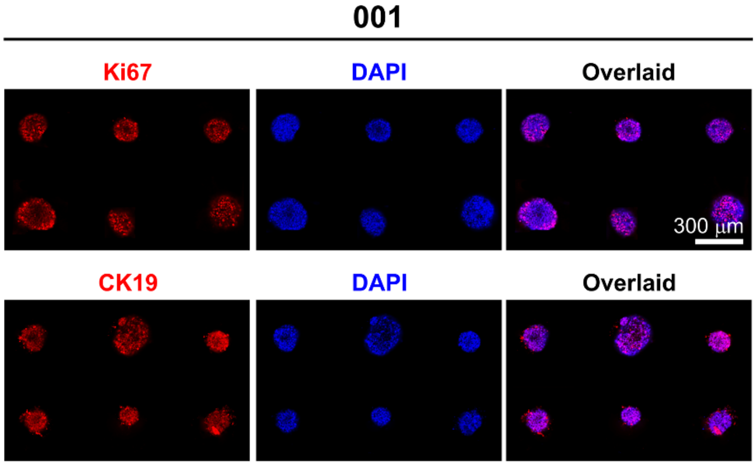

Figure S7. Immunofluorescence staining of microfluidic intact organoid cultures. Cancer specific (Ck19) and proliferation (Ki67) markers were evaluated after 7 days of culture. Nuclei were stained with DAPI (Blue).

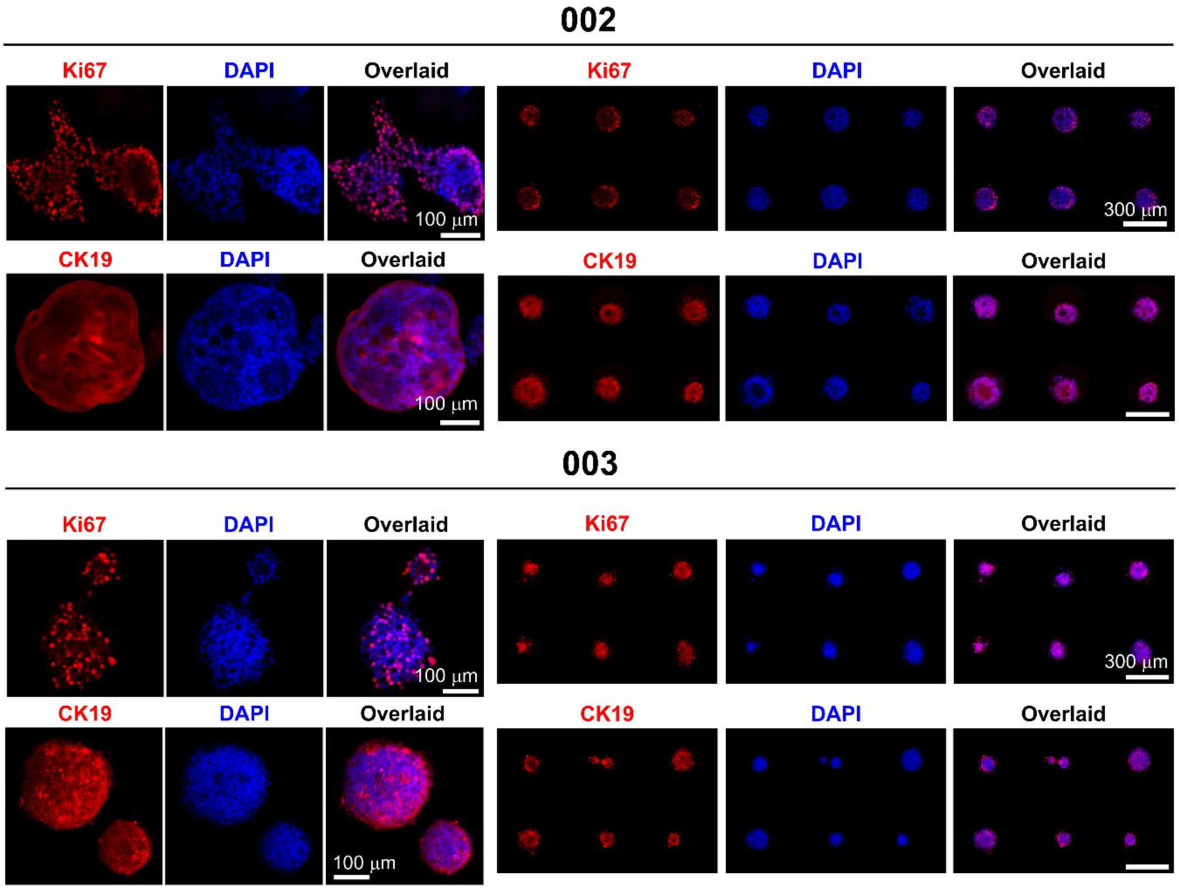

Figure S8. Immunofluorescence staining comparing Matrigel (left) and microfluidic intact organoid cultures (right) for PDO-002 and -003. Cancer specific (Ck19) and proliferation (Ki67) markers were evaluated after 7 days of culture. Nuclei were stained with DAPI (Blue).

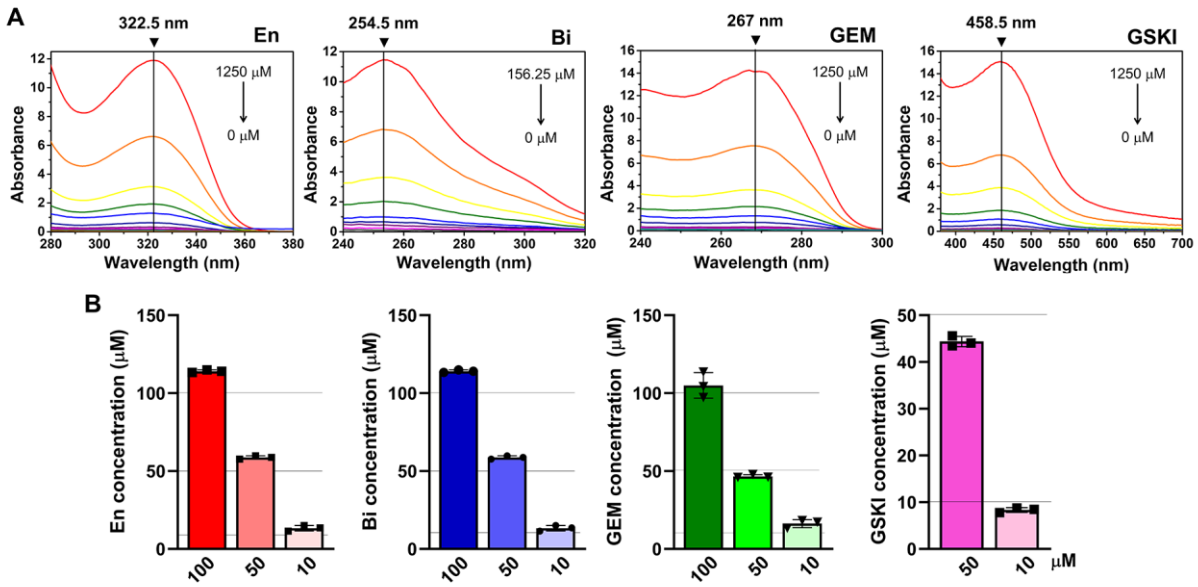

Figure S9. Characterization of drug uptake into PDMS devices. (A) UV-Visible absorbance spectra for varying concentrations of Encorafenib (En), Biminetinib (Bi), Gemcitabine (GEM) and GSKI by concentrations. Absorbance vs. concentration plots were constructed for each drug for calibration purposes. (B) Drug concentration in the media after incubation in PDMS microfluidic device for 2 days. Increased concentration is attributed to evaporation of media during incubation.

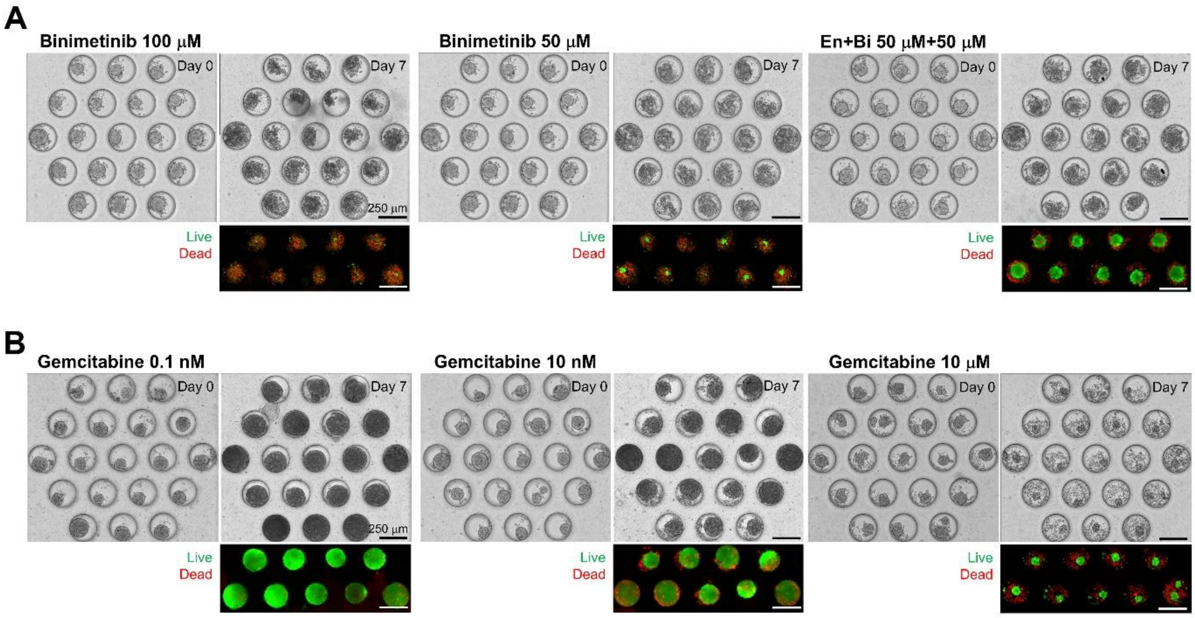

Figure S10. (A) Drug response of PDAC in μ-wells at high concentrations of Bi and En+Bi combination treatment and (B) 3 different GEM concentrations. The live/dead staining images were analyzed 7 days after drug treatment.

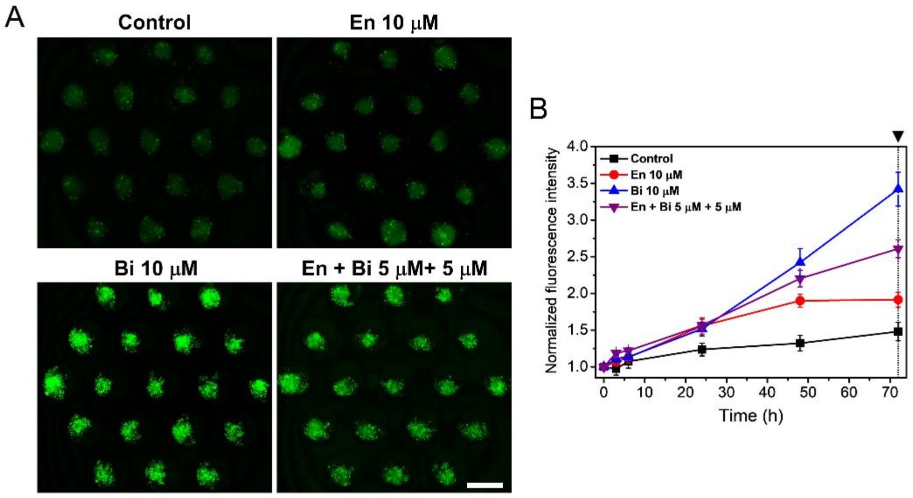

Figure S11. Assessing apoptosis in microfluidic organoids during drug testing . (A) Fluorescent detection of apoptotic cells at 48 hours after drug treatment. Control group indicates non-treated PDAC. (B) Real-time apoptotic cell intensity for En- or Bi-treated PDAC in m-wells.

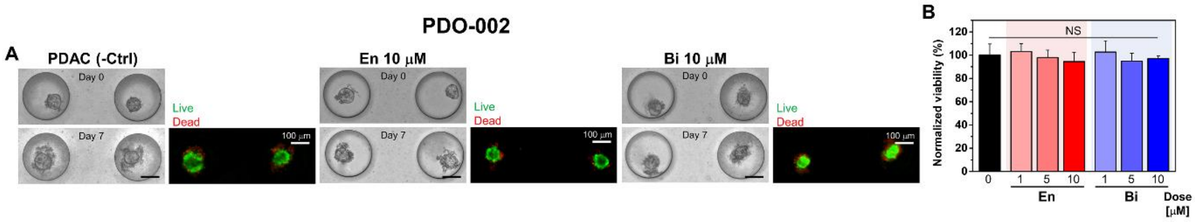

Figure S12. Assessing therapy response of BRAF wild type PDOs. (A) Images of organoids after 0-day or 7-day treatment with drugs. Live/Dead staining performed on day 7 to quantify response to therapy. Intact organoids were seeded into device type 2.(B) Viability of PDO-002 after 7 days of drug treatment that is calculated based on the Live/Dead staining.

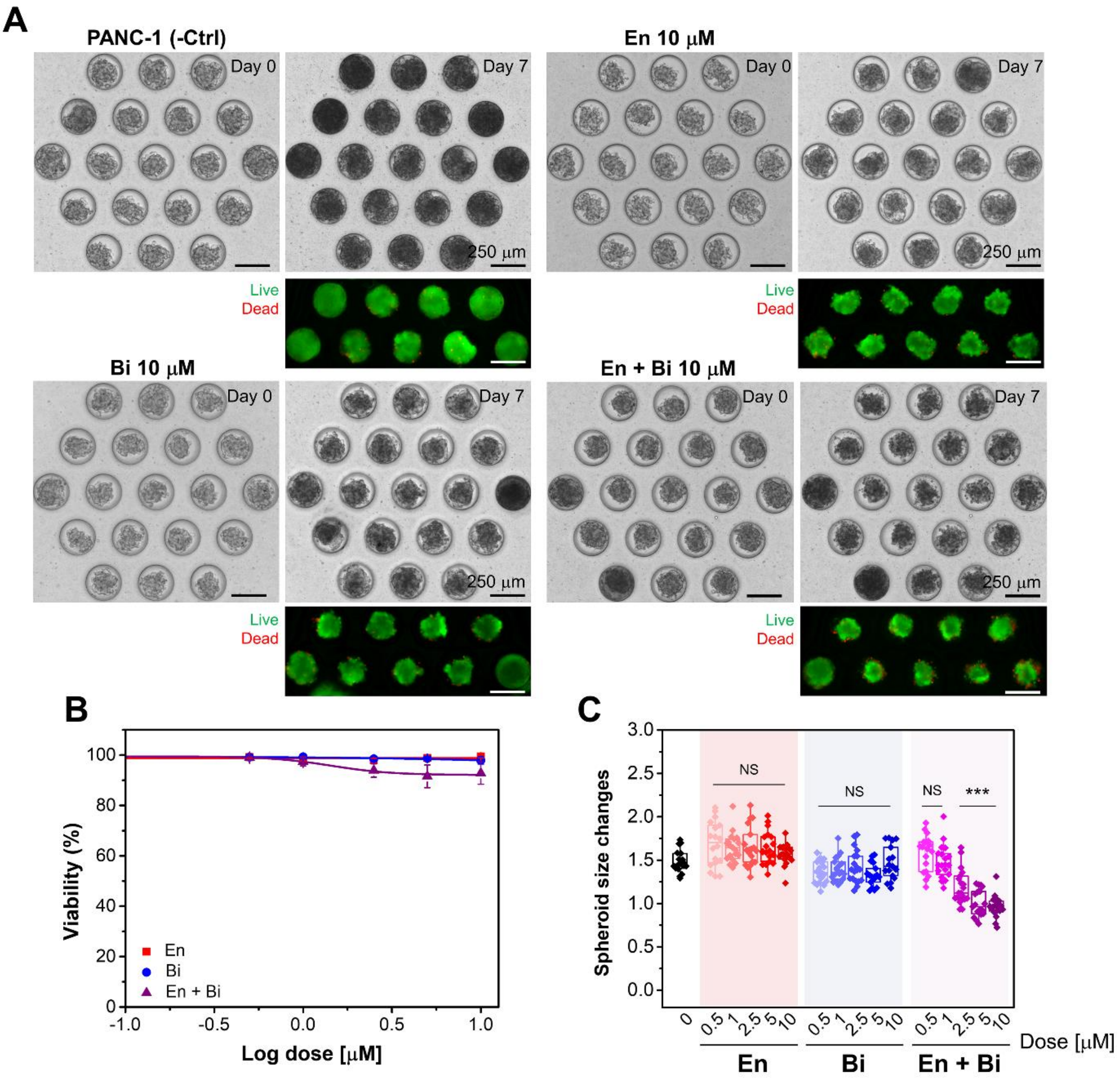

Figure S13. Assessing therapy response of BRAF wild type pancreatic cancer cell line. (A) PANC-1 in microfluidic device images before and after drug treatment. The Live/Dead staining images at day 7. (B) Dose response of PANC-1 against En and Bi therapy based on the Live/Dead staining results. (C) Spheroid size changes at day 7 compared to day 0 for all drug treatment groups. Statistically significant differences compared to control condition (0 μM) were *** $p < 0.001$. NS is not significant ($p \geq 0.05$). We have used direct single cell seeding device (device 1) to perform this experiment.

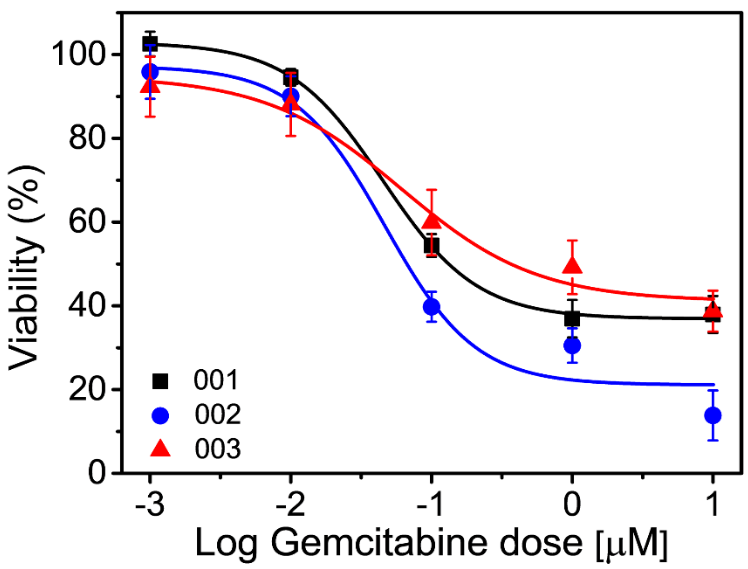

Figure S14. Dose response to Gemcitabine for microfluidic organoid cultures from 3 patients.

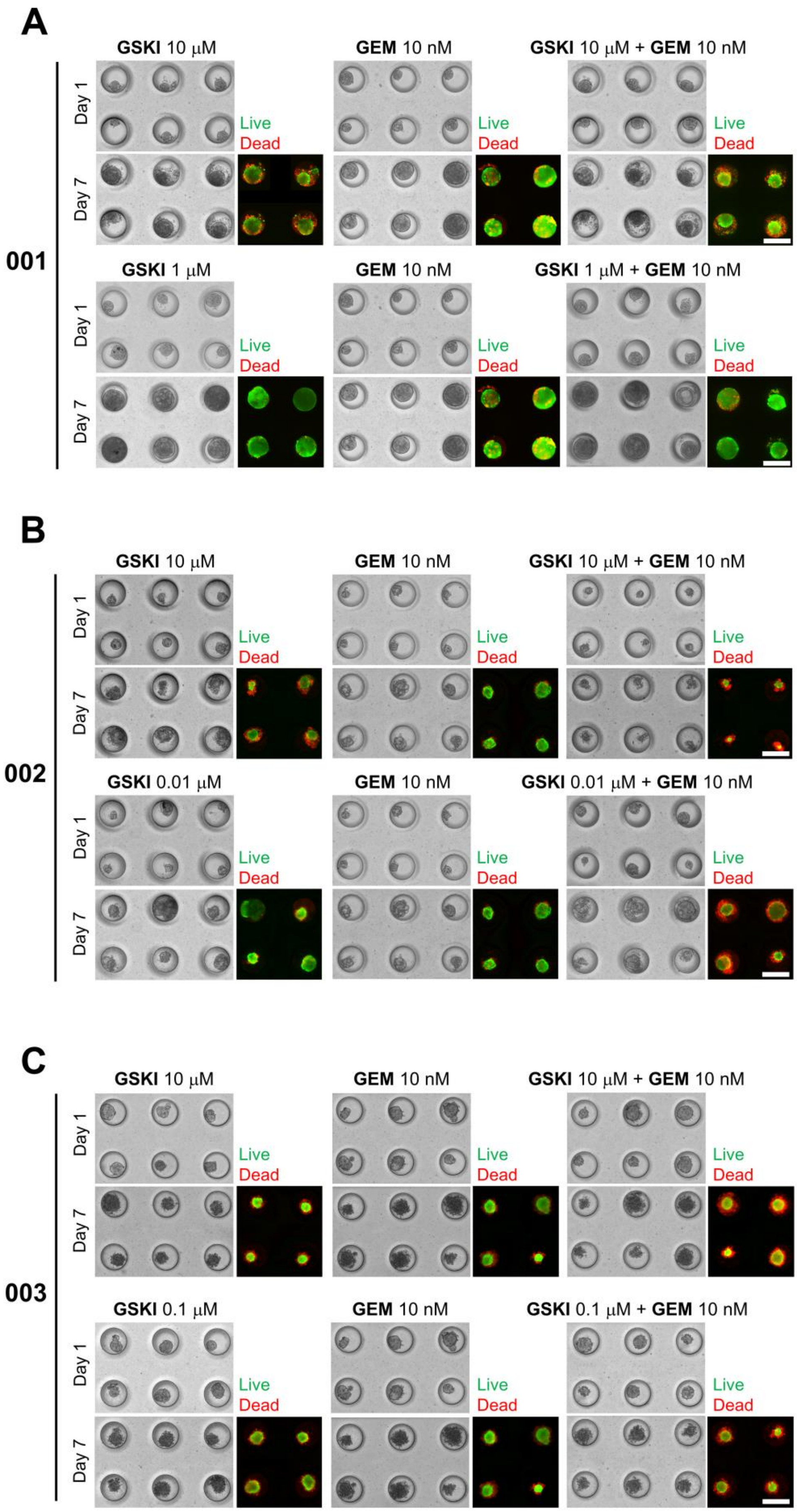
A
GSKI 10 μM
GEM 10 nM
GSKI 10 μM + GEM 10 nM
Day 1
Day 7
Live
Dead
001
GSKI 1 μM
GEM 10 nM
GSKI 1 μM + GEM 10 nM
B
GSKI 10 μM
GEM 10 nM
GSKI 10 μM + GEM 10 nM
002
GSKI 0.01 μM
GEM 10 nM
GSKI 0.01 μM + GEM 10 nM
C
GSKI 10 μM
GEM 10 nM
GSKI 10 μM + GEM 10 nM
003
GSKI 0.1 μM
GEM 10 nM
GSKI 0.1 μM + GEM 10 nM

Figure S15. GSKI and GEM combination therapy for (A) PDO-001, (B) PDO-002 and (C) PDO-003 in different concentrations. The same images for GEM 10 nM condition also appear in Figure 6B, 6D, and 6F.

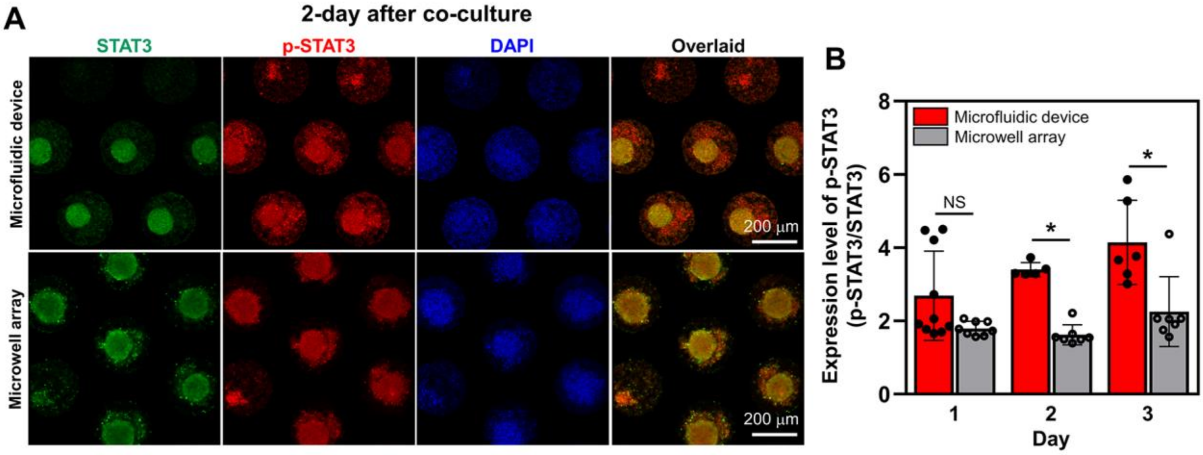

Figure S16. Evaluating interferon-γ signaling in the cancer organoid / NK cell co-cultures. (A) Immunofluorescence staining of STAT3 and phosphorylated STAT3 (p-STAT3) in NK cells and PDOs after 2 days of co-culture in the microfluidic device and the microwell array. (B) Relative expression level of p-STAT3 in both culture conditions over time. Statistically significant differences between microfluidic device and microwell were *$p < 0.05$ and NS $p \geq 0.05$ (non-significant statistically).

Table S1. List of differentially expressed and shared genes between matrigel and microfluidic devices (Excel)

Table S2. Sequences of primers for qRT-PCR analysis.

Gene	Forward	Reverse
GAPDH	TGTTGCCATCAATGACCCCTT	CTCCACGACGTACTCAGCG
CK19	CTCCCGCGACTACAGCCACT	TCAGCTCATCCAGCACCCTG
Ki67	GACAGAGGTTCCTAAGAGAG	AACAATCAGATTTGCTTCCG
Pdx1	GAAATCCACCAAAGCTCACG	CGGGTTCCGCTGTGTAAG

References

[1] a) N. Dadgar, A. M. Gonzalez-Suarez, P. Fattahi, X. Hou, J. S. Weroha, A. Gaspar-Maia, G. Stybayeva, A. Revzin, *Microsystems & nanoengineering* **2020**, *6*, 1; b) J. M. de Hoyos- Vega, H. J. Hong, K. Loutherback, G. Stybayeva, A. Revzin, *Advanced Materials Technologies* **2022**, 2201121.
